# Stochastic principles governing alternative splicing of RNA

**DOI:** 10.1371/journal.pcbi.1005761

**Published:** 2017-09-14

**Authors:** Jianfei Hu, Eli Boritz, William Wylie, Daniel C. Douek

**Affiliations:** 1 Genome Analysis Core, Vaccine Research Center, National Institute of Allergy and Infectious Diseases, National Institutes of Health, Bethesda, Maryland, United States of America; 2 Human Immunology Section, Vaccine Research Center, National Institute of Allergy and Infectious Diseases, National Institutes of Health, Bethesda, Maryland, United States of America; Rutgers University, UNITED STATES

## Abstract

The dominance of the major transcript isoform relative to other isoforms from the same gene generated by alternative splicing (AS) is essential to the maintenance of normal cellular physiology. However, the underlying principles that determine such dominance remain unknown. Here, we analyzed the physical AS process and found that it can be modeled by a stochastic minimization process, which causes the scaled expression levels of all transcript isoforms to follow the same Weibull extreme value distribution. Surprisingly, we also found a simple equation to describe the median frequency of transcript isoforms of different dominance. This two-parameter Weibull model provides the statistical distribution of all isoforms of all transcribed genes, and reveals that previously unexplained observations concerning relative isoform expression derive from these principles.

## Author summary

Alternative RNA splicing within eukaryotic cells enables each gene to generate multiple different mature transcripts which further encode proteins with distinct or even opposing functions. The relative frequencies of the transcript isoforms generated by a particular gene are essential to the maintenance of normal cellular physiology; however, the underlying mechanisms and principles that govern these frequencies are unknown. We analyzed the frequency distribution of all transcript isoforms in highly purified human T cell subsets and built a simple mathematical model, based on the physical process of alternative splicing, which provides statistical principles that govern this process. This model matches very well with the observed distributions of expression levels and relative frequencies of all transcript isoforms from different tissues and cell lines. Notably, we used this model to elucidate many previously unexplained observations concerning transcript isoform expression. More importantly, this model reveals the existence of simple statistical principles that can be applied to understanding an essential and complex biological process such as alternative splicing.

## Introduction

Most genes of eukaryotic organisms, unlike those of prokaryotes, may each generate multiple different mature transcript isoforms which can encode proteins with distinct or even opposing functions [1–6]. It has also been shown that the dominance of the major transcript isoform from a single gene may radically affect cell function, identity and fate, and that disruption of this dominance may result in human disease, including abnormal osteoclast genesis, tumorigenesis and Parkinson’s disease [3–11]. In addition, three intriguing observations have been reported regarding the frequency distribution of transcript isoforms that point to universal principles governing gene transcript isoform expression: (1) genes tend to express all their isoforms simultaneously but at different levels; (2) the major and minor dominant isoform of a gene usually accounts for over 30% and 15% of total transcript expression, respectively; and (3) for any two distinct transcript isoforms from the same gene, one of them is always significantly dominant [3–14]. However, the mechanisms underlying these fundamental observations remain unclear. Indeed, the overall expression and frequency distribution of all isoforms of entire transcriptomes has rarely been subjected to systematic analysis.

## Results

### A stochastic model for alternative splicing

Many studies have been performed to identify cis-acting elements, trans-acting factors and the specific biological processes involved in AS [1,15–18]. Essentially, the AS process contains two major steps: (1) intron identification by the binding of U1 and U2AF proteins to the 5’ and 3’ splice sites, respectively; and (2) intron splicing by the release of U1 and the additional binding of U4-6 snRNP [1,15–18]. We focused on intron identification as this decides the fate of the pre-mRNA by determining which transcript isoforms will be produced.

**Fig 1:**
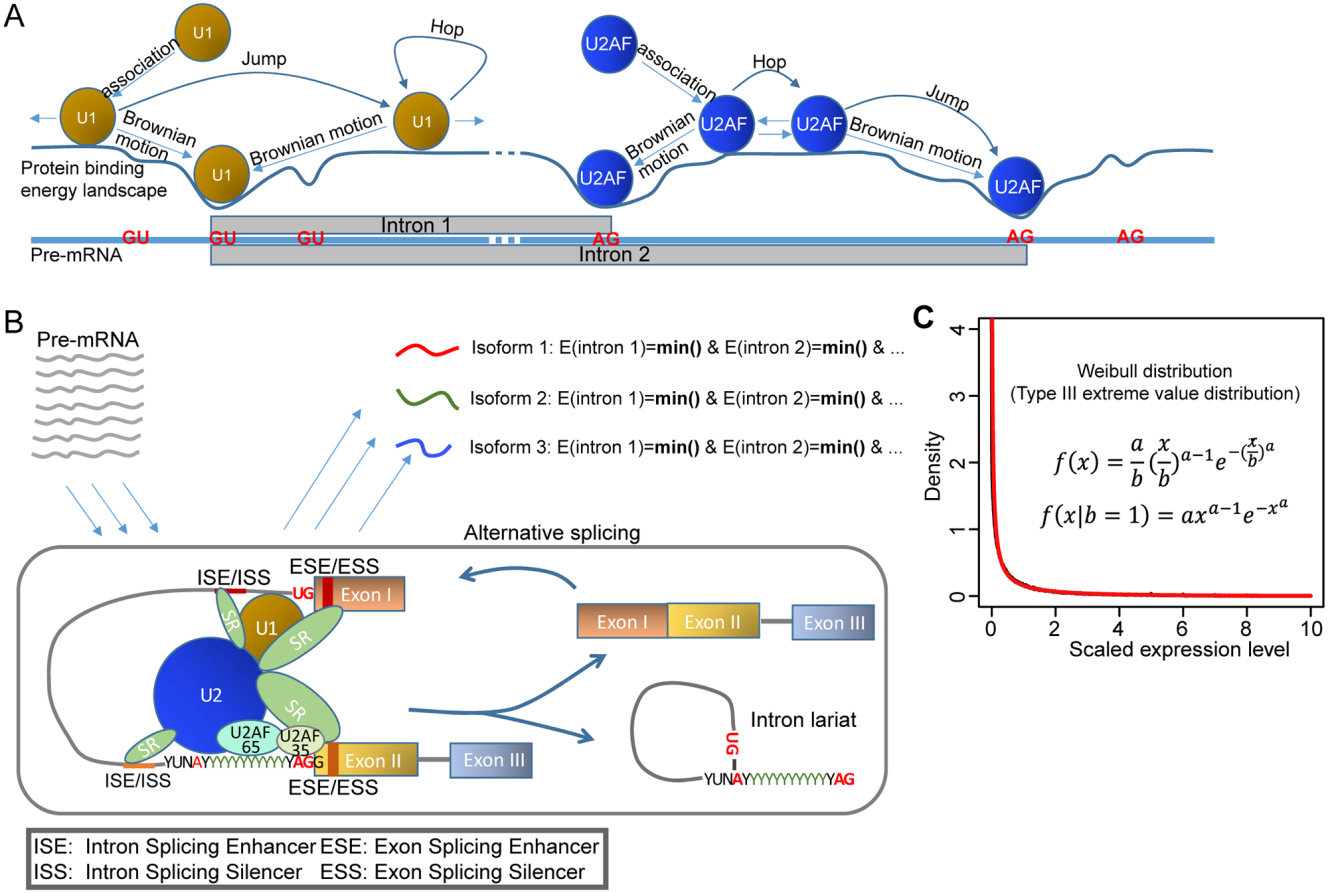
A model of alternative splicing. (A) Splicing factor U1 and U2AF search the 5’ GU and 3’ AG splicing sites by 3D and 1D Brownian motion. Multiple candidate splice sites compete for the binding of U1 and U2AF. The binding is ATP-independent and reversible. (B) The binding of U1 and U2AF to the splice sites becomes stable only after the ATP-dependent binding of U2 snRNP. The identification of each intron is equivalent to a minimization process that U1 and U2AF dynamically search their global or local minimal energy sites on the pre-mRNA segment presented for AS. (C) The scaled expression level of transcript isoform follows type III extreme value distribution—a Weibull distribution. The approximate values of parameters *a (0*.*44)* and *b (0*.*6)* are estimated by curve fitting. Black curve represents the distribution of scaled expression level from experimental data. Red curve represent the Weibull distribution produced by curve fitting.

U1 and U2AF engage in random three-dimensional (association/dissociation) and one-dimensional (sliding) Brownian search (Fig 1A) [19,20]. The binding of U1 and U2AF to the splice sites is ATP-independent, weak and reversible, and becomes stable only after the ATP-dependent binding of U2 snRNP (Fig 1B) [21]. Usually, in a segment of pre-mRNA presented for AS, many candidate splice sites exist and compete for the binding of U1 and U2AF. The lower the potential energy of the splice sites, the stronger the binding and the more time allowed for the formation of a stable A-complex, the more corresponding mature mRNA will be produced. This indicates that the process of AS is stochastic: the product of a transcript isoform from a pre-mRNA is probabilistically determined by the binding energy of splicing factors at splice sites. Mathematically, this process represents a stochastic minimization process in which U1 and U2AF dynamically search their global or local minimal potential energy sites on the pre-mRNA segment since non-minimal potential energy sites are not stable thus don’t have enough time to allow the formation of stable A-complex. A mature mRNA will undergo multiple rounds of the minimization process if the corresponding pre-mRNA has multiple introns to remove (Fig 1B). This suggests that the expression levels of transcript isoforms may follow an extreme value distribution, of which there are only three types whatever the original distribution of the random variables; namely, Gumbel distribution (Type I), Frechet distribution (Type II) and Weibull distribution (Type III). These three distributions can be transformed to each other by a simple mathematical transformation of the original random variable [22–24].

We tested the three candidate distributions by performing whole transcriptome deep sequencing (RNA-seq) on highly purified resting and activated peripheral blood human CD4 T cell subsets (naïve, central memory, transitional memory and effector memory) from 9 healthy donors (S1 Table). Because the AS mechanism is the same for all multiple-exon genes, the distribution function that their transcript isoforms follow should be similar. However, certain parameters of the distribution function may differ according to the level of expression of a gene as determined by the activation state of the cell. Thus, a scaling parameter must be applied to the raw expression levels of transcript isoforms from different cell conditions and genes. A simple scaling factor is the average expression level of all transcript isoforms from the same gene, as it positively correlates with the gene’s expression level but is independent of isoform number. The smaller the isoform number *M*, the greater the inaccuracy in the estimation of the scaling factor, and, for accuracy, only genes with *M*≥5 are used here. Our analysis reveals that the expression levels of transcript isoforms of a gene follows a type III Weibull extreme value distribution—*W(x*,*a*,*b)* (Fig 1C).

$$W\left(x\right)=W\left(x,a,b\right)=\frac{a}{b}{\left(\frac{x}{b}\right)}^{a-1}e^{-{\left(\frac{x}{b}\right)}^{a}}$$ (1)

*W(x)* is the probability of a transcript isoform with expression level x; *b* is the scale parameter, which will change with the expression level of gene; and *a* is the shape parameter, which is specific to the AS mechanism and should be constant for all genes. The approximate values of parameters *a (0*.*44)* and *b (0*.*6)* in Fig 1C are estimated by simple curve fitting, and thus are somewhat inaccurate.

For the Weibull distribution, a simple formula links *a*, *b* and the population mean of μ [24,25],

$$\mu =b\Gamma \left(1+\frac{1}{a}\right)$$ (2)

Γ represents Gamma function. For a gene with *M* transcript isoforms and expression level of *E*, the sample mean $\bar{x}=E/M$. When the isoform number *M* is sufficiently large, the sample mean approaches the population mean, giving an approximate formula that connects transcript isoform number *M*, gene expression level *E* and two parameters *a* and *b*,

$$E\approx bM\Gamma \left(1+\frac{1}{a}\right)$$ (3)

Of note, the analysis above shows that the correct scale factor *b* should be $\bar{x}/\Gamma \left(1+\frac{1}{a}\right)$, not $\bar{x}$.

Although the experimental data fit very well with the Weibull distribution empirically, the statistical test of the fitness-of-fit is not significant for four reasons. First, our model is a very simple one, which considers only the most important factor influencing AS, the strength of splice site binding, and disregards many other factors such as co-transcriptional splicing, histone modifications on chromatin, poison exons, non-sense mediated degradation (NMD) and so on; Second, there is bias in the estimation of the scale factor, and furthermore this bias changes with isoform number *M*. Third, it is well known that current annotations for human transcript isoforms are incomplete; thus, the transcript isoform number *M* used for many genes is not accurate. Fourth, although significant improvement has been made in the accuracy of calculation of transcript isoform expression levels, current algorithms nevertheless remain imperfect.

### The statistical distribution of the frequencies of all transcript isoforms

**Fig 2:**
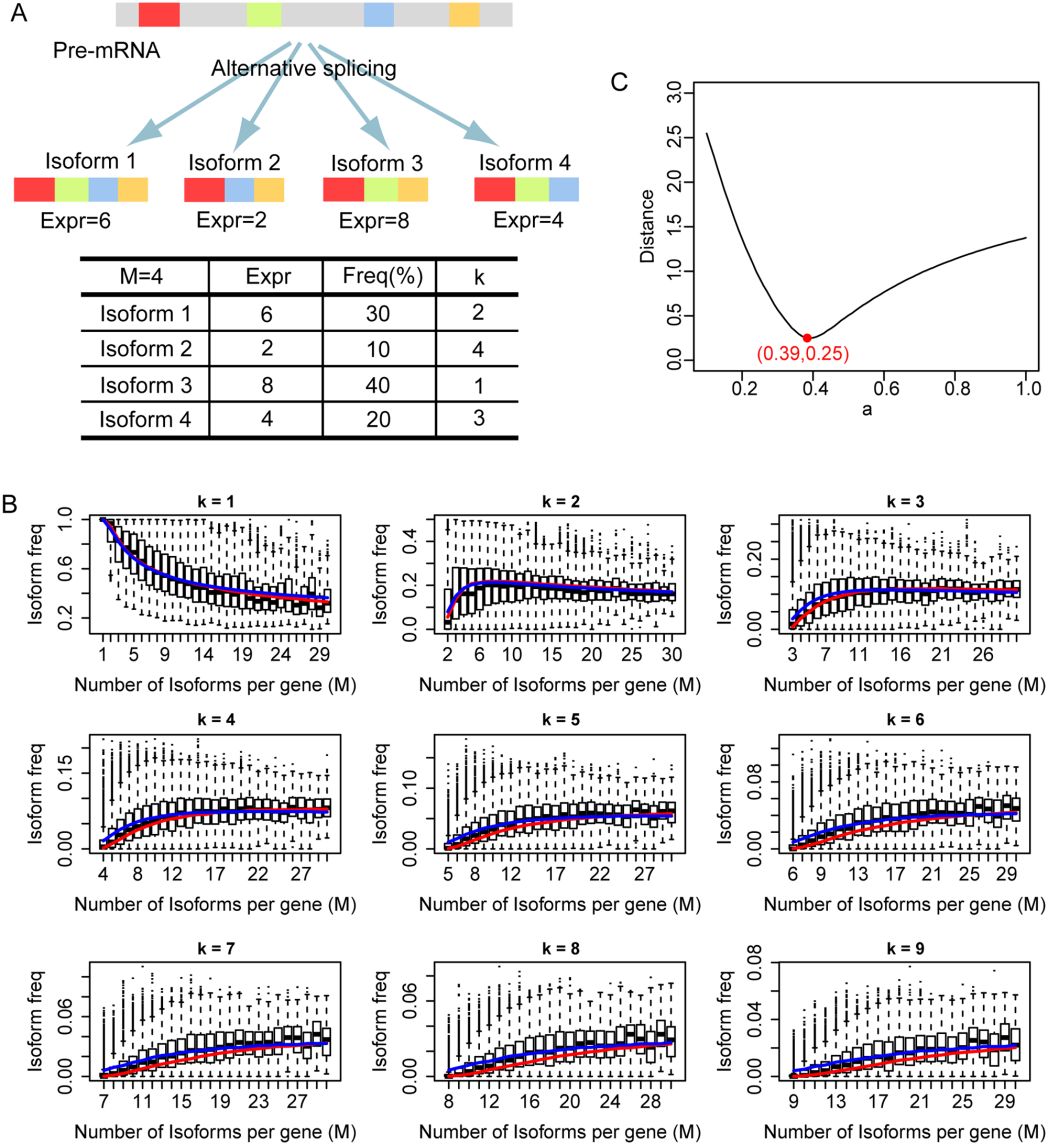
The frequency distribution of transcript isoforms. (A) Schematic diagram of alternative splicing and calculation of transcript isoform frequencies. Colored regions represent exons. Gray regions represent introns and intergenic sequences. For simplification, the expression values of isoforms are taken as integers. (B) The boxplot distribution of transcript isoform frequency *f(k*, *M)* with fixed *k* and increasing *M*. *k* is the rank of transcript isoform. *M* is the number of transcript isoforms of genes. Boxplot represents frequency distribution calculated from our RNA-seq data by Cufflinks based on merged gene datasets. Blue curve represents median values calculated from the approximation formula (4). Red curve represents median values from simulation of Weibull distribution *W(0*.*39)*. (C) The distribution of the Euclidian distance relative to different *a* for all *mf(k*,*M)* in Fig 2B between experimental data and simulated data from Weibull distribution. The distance reaches the minimum when *a* = 0.39.

We defined the frequency of a gene’s transcript isoform as the ratio of its expression level relative to the expression level of the gene, which equals the sum of expression levels of all transcript isoforms from that gene (Fig 2A). Thus, for a gene with *M* different transcript isoforms where each isoform has the rank *k* in the hierarchy of expression levels, we use *f(k*, *M)* to represent the frequency of the *k*th dominant isoform. As 1≤*k*≤*M*, so *f(1*, *M)* ≥ *f(2*, *M)* ≥ … ≥ *f(M*, *M)*. *f(k*, *M)* was entirely stochastic, differed among genes and changed with cell activation state, except for *f(1*,*1)* which was always 100% as long as the corresponding gene was expressed. For example, *f(1*,*2)*—the frequency of the most dominant isoform of a gene with two transcript isoforms—varied between 50% and 100%. *f(2*,*2)*—the frequency of the second most dominant isoform—varied between 0 and 50%. For a specific gene, both the frequencies and the ranks of its isoforms may change with cell condition, such that the most dominant isoform of a gene in one condition may become a less dominant isoform in other conditions. Thus, for the same gene (same *M*) and same *k*, *f(k*, *M)* may represent the frequency of a different isoform under different cell conditions. While notable, this property is inconsequential as the following analyses explore the relationship solely between the frequency and rank of transcript isoforms. To analyze the frequency distribution of transcript isoforms, we grouped genes according to the number of their isoforms from group 1, which contains genes with one isoform, through group *M* which contains genes with *M* isoforms. The variation in isoform frequency with *k* and *M* is illustrated straightforwardly in a boxplot (Fig 2B) which shows that the frequency of the most dominant isoform decreases with *M*. This trend becomes more apparent if we focus solely on the median value, *mf(k*,*M)*. The median frequency of the most dominant isoform *mf(1*,*M)* decreases from 100% when *M* = 1, to approximately 50% when *M* = 10, and approximately 30% when *M* = 30. In contrast, the median frequency of the second most dominant isoform *mf(2*,*M)* initially increases, peaking when *M* = 6, and then decreases with *M*. The median frequencies of other isoforms *(k>2)* show a similar trend. These results confirm and extend a previous report where only the most dominant isoform *(k = 1)* was analyzed [13].

Notably, our model was not only able to explain and provide the overall distribution of the scaled expression levels of all transcript isoforms, it could also provide the frequency distribution of all transcript isoforms. We may show this using a Monte Carlo simulation. The scale parameter *b* was set as 1 as it has no influence on this simulation of transcript isoform frequency.

First, we showed that all *mf(k*,*M)* can be explained by our model and, in the process, also showed how *mf(k*,*M)* could give a more accurate estimation of the shape parameter *a*—approximately 0.44. We randomly selected a number in the range (0, 1) as the value for *a* and performed the following computation: for genes with *M* transcript isoforms, we randomly extracted *M* numbers from the Weibull distribution *W(a*,*1)* as the expression levels of the *M* simulated isoforms, which can then be transformed to their frequencies. We repeated this process 10,000 times for each *M* to obtain the simulated median frequency *mf(k*,*M)* and then compared it with the corresponding *mf(k*,*M)* from our experimental data. The Euclidian distance of all *mf(k*,*M)* from simulated data and experimental data in Fig 2B was calculated and reached the minimal value when *a* was 0.39 (Fig 2C). Notably, 0.39 is also the exact solution of equation 1+1/a = Γ(1+1/a). Fig 2B shows that when *a* = 0.39, values for *mf(k*,*M)* calculated from the simulated data (red curves) are very close to those from the experimental data (box plot). The shape parameter *a* calculated by the Monte Carlo simulation is more accurate than that calculated by simple curve fitting for two reasons. First, the median value of a distribution is very stable, and sampling error and outliers has relatively less influence on its estimation. Second, the bias in the estimation of scale factor is same for both the experimental and simulated datasets and thus its influence is canceled out. To help understand how *mf(k*,*M)* changes with *a*, similar figures with *a* = 0.2 and *a* = 0.6 are also given in the supplemental material (S1 Fig). Fig 2B reveals that the median frequency of the most dominant isoform, *mf(1*, *M)*, decreases with *M* and has no lower limit. This finding contradicts a previous observation that the frequency of the most dominant isoform is at least 30%, even for a gene with many isoforms [13].

**Fig 3:**
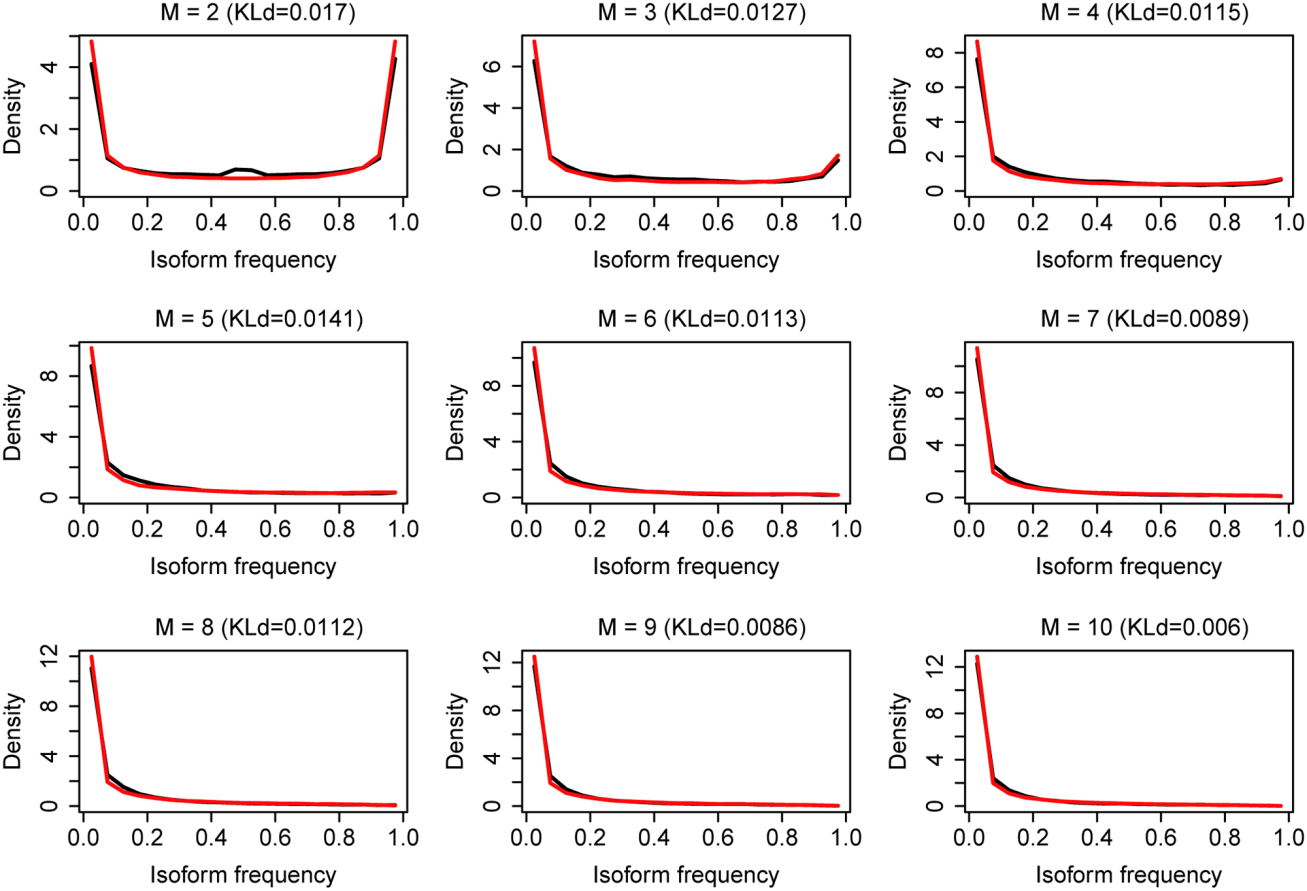
The frequency distribution of all transcript isoforms from experimental data and simulated data for *M* = 2:10. *M* is the number of transcript isoforms for a gene. Black curves represent experimental data, red curves represent simulated data from *W(0*.*39)*. KLd is the Kullback-Leibler divergence between the two distributions.

**Fig 4:**
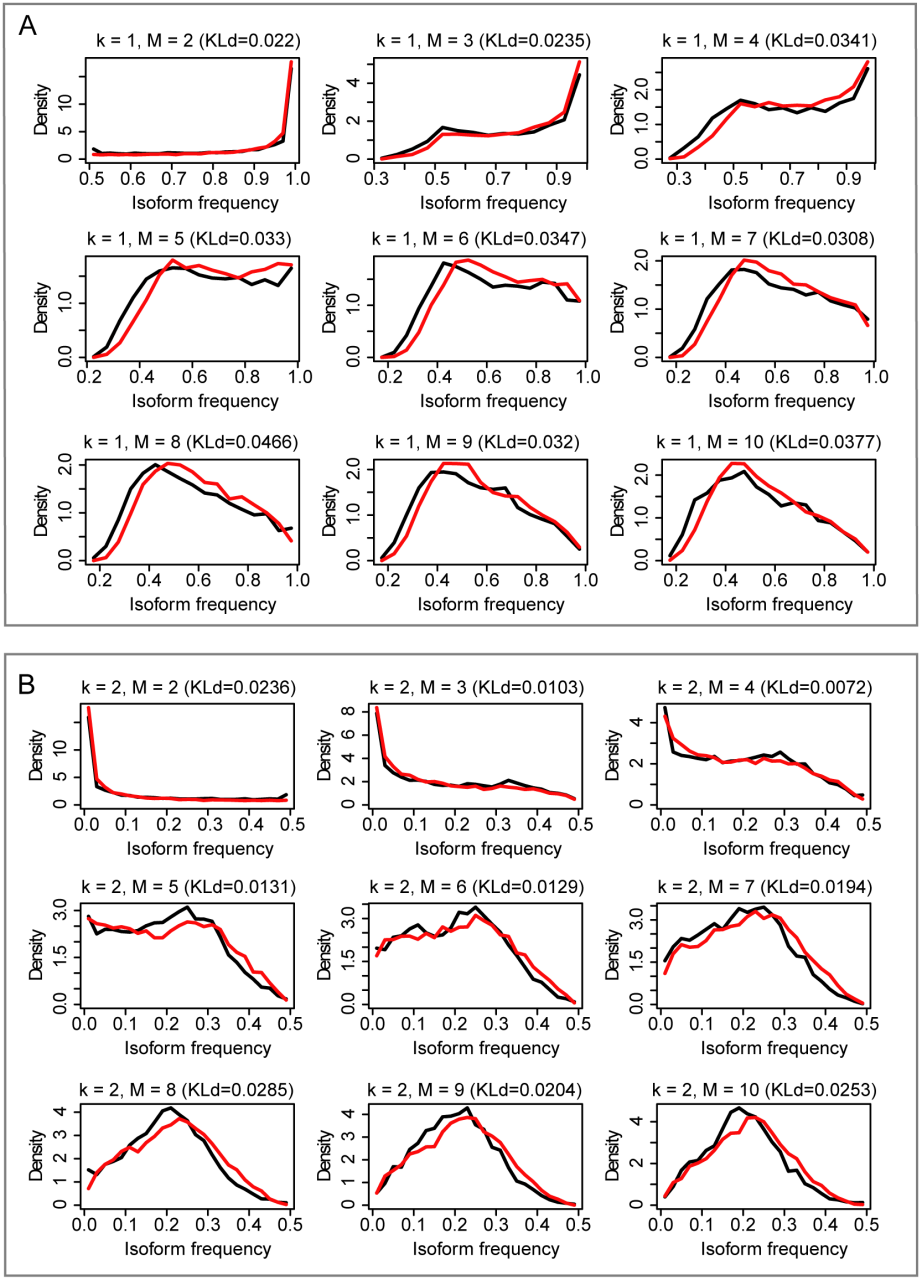
The frequency distribution of the *k*th dominant transcript isoform. (A) *k* = 1. (B) *k* = 2. *k* is the rank of a transcript isoform. *M* is the number of transcript isoforms for a gene. Black curves represent frequency distribution of the experimental RNA-seq data. Red curves represent the frequency distribution of the simulated data from Weibull distribution *W(0*.*39)*. KLd is the Kullback-Leibler divergence between the two distributions.

Second, we showed that the frequency distribution of all transcript isoforms as well as each *f(k*,*M)* can be given by our model. Repeating the previous Monte Carlo simulation with *a* = 0.39, we obtained the frequency distribution of all transcript isoforms for different gene groups (different M) (Fig 3) and each *f(k*,*M)* (Fig 4 and S2 Fig) from the simulated data. Here, we use Kullback-Leibler divergence (KLd) to evaluate the difference between the two distributions, which represents the amount of information lost when we used the simulation of our Weibull model to represent the frequency distribution of the experimental data. We found that for most frequency distributions analyzed, the amount of information lost is smaller than 0.05 (mean = 0.026, median = 0.020). This shows that the frequency distribution from the simulated data (red curve) is highly consistent with that from the experimental data (black curve), although the shape and range of the distribution change with *k* and *M*. Thus, although the expression levels of transcript isoforms change with a particular gene, cell condition and rank, their overall frequency distributions do not change and can be described by our model.

Third, these distributions enable statistical analysis of transcript isoform usage such as defining significantly dominant transcript isoforms. For genes with two, five, ten, 20 and 30 transcript isoforms, an isoform may be called significantly dominant if its frequency is above 0.99, 0.852, 0.529, 0.269 and 0.174, respectively, since the probability that an isoform randomly selected has frequencies above these thresholds is less than 5% (S2 Table). Thus, the ability to define such thresholds may be used as a statistical framework to discover functionally dominant transcript isoforms with relevance to disease states.

### A simple formula for the frequency distribution of transcript isoforms

The frequency distribution of transcript isoforms changes with their rank *k* and the isoform number *M* of the gene from which they are spliced. Nonetheless, our model was able to give the distribution of each *f(k*,*M)* as well as their median frequency *mf(k*,*M)*. Remarkably, we also found that all *mf(k*,*M)* could be described by a simple formula:

$$mf\left(k,M\right)=\frac{1/\left(\frac{k}{M}\times e^{{\left(1+\frac{k}{M}\right)}^{2}}\right)}{\sum_{m=1}^{M}1/\left(\frac{m}{M}\times e^{{\left(1+\frac{m}{M}\right)}^{2}}\right)}=\frac{\frac{1}{k}\times e^{-{\left(1+\frac{k}{M}\right)}^{2}}}{\sum_{m=1}^{M}\frac{1}{m}\times e^{-{\left(1+\frac{m}{M}\right)}^{2}}}=\frac{e^{-{\left(1+\frac{k}{M}\right)}^{2}}}{k\times H_{M}}$$ (4)

Here, *H*_*M*_ is the *M*_*th*_ generalized harmonic number:

$$H_{M}=\sum_{m=1}^{M}\frac{1}{m}\times e^{-{\left(1+\frac{m}{M}\right)}^{2}}$$ (5)

Fig 2B shows that the median frequencies computed by the formula above (blue curves) are very close to the values from the experimental data and simulated data across all values of *k* and *M*, indicating that the median frequency of the *k*th dominant isoform of a gene with *M* isoforms is proportional to $\frac{1}{k}\times e^{-{\left(1+\frac{k}{M}\right)}^{2}}$, which thus can be taken as its frequency index.

### Verification of the computational approach

Four important methodological points were addressed. First, to exclude the possibility that our results emerged from an intrinsic property of the analysis software, we reanalyzed the entire dataset with an independent software package, Salmon [26,27], which, in contrast to Cufflinks, requires no sequence alignment. The similarity of the results derived from these two approaches indicates that the isoform frequency distribution we observed is robust and software-independent (S3 Fig). Second, to exclude the possibility that our results emerged from the Expectation Maximization (EM) algorithm used by most software packages, we created two simulated RNA-seq datasets with transcript isoform expression level following a Normal distribution N(20,2) and a Weibull distribution W(0.39,10), respectively (See Materials and methods for details). The results based on the simulated RNA-seq dataset from the Normal distribution were markedly different from those from our experimental RNA-seq data (S4 Fig). In contrast, the results based on the simulated RNA-seq dataset from the Weibull distribution showed a perfect match with our experimental RNA-seq data (S5 Fig). Third, to exclude the possibility that our results emerged purely as a function of the particular dataset we used, we analyzed 18 different pre-existing RNA-seq datasets derived from embryonic stem cells, cancers and human cell lines (S3 Table). We obtained similar results in every case (S6 and S7 Figs). Finally, the result based on a merged gene set (Euclidian distance 0.158) showed a closer match with our formula than the result based solely on the Ensembl gene set (Euclidian distance 0.160; S8 Fig), which reflects the incomplete nature of existing datasets.

The correctness of our model is strongly supported by two points. First is the simplicity of the model in that it requires only one shape parameter. Second is its general applicability in explaining the scaled expression level of all transcript isoforms, the frequency distribution of transcript isoforms of genes with different isoform number (*M*), and furthermore the frequency distribution of individual transcript isoforms of different rank (*k*). Here, we did not perform a strict mathematical deduction of how stochastic searching of minimal-energy U1 and U2AF binding sites leads to the Weibull distribution as this would be extremely difficult if not impossible, as Weibull himself discussed in his original paper: “it is utterly hopeless to expect a theoretical basis for distribution functions of random variables such as strength properties of materials or of machine parts of particle size” [25]. Currently, our model takes the isoform annotation of all genes given by the user as input (we recommend the latest Ensembl transcript annotation), and it does not explain or predict the isoform number of genes.

### The dominancy rank of transcript isoforms can be regulated by external signals

**Fig 5:**
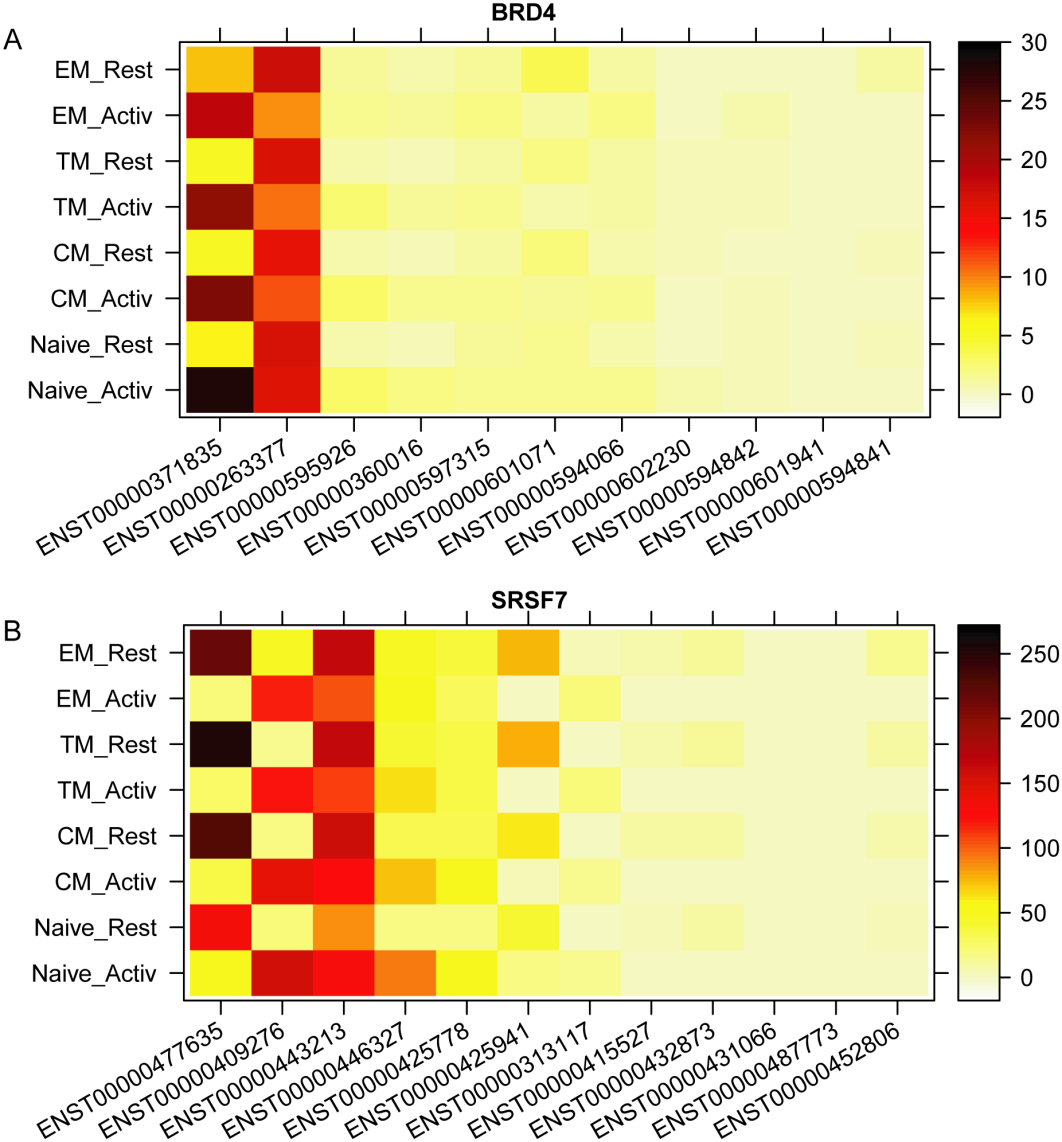
Transcript isoform expression pattern of two genes in different conditions. (A) BRD4. (B) SRSF7. Among 11 transcript isoforms of BRD4 and 12 transcript isoforms of SRSF7, ENST00000371835 and ENST00000409276 are the most dominant isoforms in all four activated conditions, ENST00000263377 and ENST00000477635 are the most dominant isoforms in all four resting conditions, respectively. This result indicates the major transcript isoform can be regulated by single external signal.

It should be noted that the binding potential energy landscape of U1 and U2AF on a specific pre-mRNA segment is not static but dynamic, and may change with the binding of other tissue-specific or non-specific auxiliary proteins on cis-acting AS elements induced by external signals; thus, genes may have different major transcript isoforms under different conditions. We analyzed the change of dominancy rank of transcript isoforms for every expressed gene in the four T cell subsets under the two cell conditions: resting and after in vitro activation. Using the Ensembl gene set, 540 genes underwent transformation of the most dominant transcript isoforms between resting and activated conditions across all four T cell subsets, another 891 genes underwent transformation of the most dominant isoform for three of the four T cell subsets (S4 Table). The biological processes enriched in the 540 genes are very diverse and include regulation of cellular response to stress, virus-host interaction, chromosome organization, transcription, translation and protein metabolism (S9 Fig). This suggests that a T cell may express not only different genes but also different transcript isoforms depending on its activation state. For example, BRD4 (bromodomain containing 4), an inhibitor gene of HIV-1 infection [28], has 11 known transcript isoforms. Of them, ENST00000371835 is the most dominant isoform in the activated condition of all four T cell subsets and the second most dominant in the resting condition, while ENST00000263377 is the most dominant isoform in the resting condition in all four T cell subsets and the second most dominant in the activated condition (Fig 5A). SRSF7 (serine/arginine-rich splicing factor 7), a splicing factor and inhibitor of HIV-1 Tat-mediated transactivation [29], has 12 distinct transcript isoforms. Of them, ENST00000409276 is the most dominant transcript isoform in the stimulated condition across all four T cell subsets, whereas ENST00000477635 is the most dominant transcript isoform in resting condition across all four T cell subsets (Fig 5B). Taken together, these and our previous results demonstrate that the dominancy rank of transcript isoforms of a gene can be regulated by external stimuli, but that the frequency distribution of transcript isoforms at each rank remains constant.

### The number of transcript isoforms expressed versus those annotated

**Fig 6:**
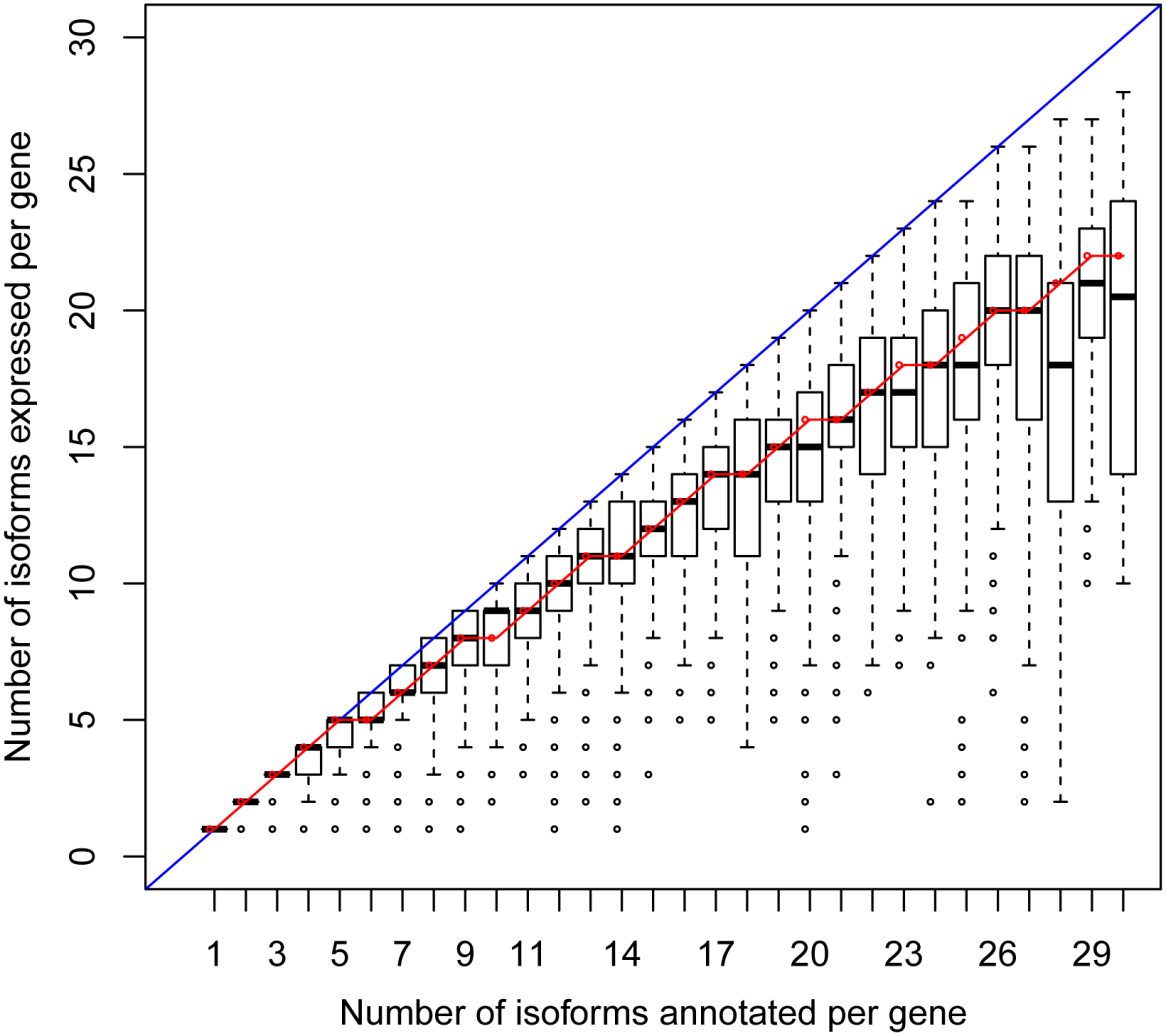
The number of isoforms expressed versus those annotated. The boxplot is the observed result from our RNA-seq data. The red curve is the expected median calculated from our Weibull model.

It has been reported that the number of isoforms expressed increases with the number of isoforms annotated per gene [13]. We redid the analysis with our own RNA-seq data and confirmed these findings and, more importantly, can provide an explanation. To calculate the expected number of isoforms expressed, we still used the expression level of transcripts from the simulated Weibull distribution W(0.39). Different genes have different expression levels; thus it is reasonable to select a cutoff of frequency rather the absolute expression level to define whether a transcript isoform would be theoretically detected. Here, we use 0.001 as the frequency cutoff and thus define undetectable transcript isoforms as those whose frequency is below 0.001. The boxplot is the observed result from our RNA-seq data (Fig 6). The red curve is the expected median calculated from our model guided by two assumptions: 1) genes express all their transcript isoforms simultaneously; 2) the scaled expression level of transcript isoform follows W(0.39). There is excellent concordance between the two plots, and they both show that the number of isoforms expressed increases with the number of transcripts annotated per gene.

### The stochastic model of AS provides a mechanism for previously unexplained observations

Our model may be applied to a number of key observations that have been made in previous studies on the usage of AS transcript isoforms but for which mechanistic explanations have been lacking. The first observation is that genes tend to express all their transcript isoforms simultaneously but at different levels [13]. We can explain this mathematically because the Weibull distribution, when *a*<1 (here *a* = 0.39), peaks at 0 and then decreases at a rate greater than an exponential distribution. Thus the expression level of most transcript isoforms will be slightly higher than 0, while the expression level of the remaining transcript isoforms will be considerably higher than 0 and will differ from each other. The second observation is that the major and minor isoforms of a gene usually account for over 30% and 15% of total transcript expression, respectively [12–14]. The theoretical percentage of human genes with *f(1*,*M)* ≥ 30% can be calculated from the weighted number of genes in each gene group. We first calculated the percent of simulated genes with *f(1*,*M)* ≥ 30% for each gene group in the simulated dataset and then used the result to weight the number of human genes in that group. The result revealed that 93% of genes that undergo AS will have *f(1*,*M)*>30%. Similar analysis revealed that 60% of genes that undergo AS will have *f(2*,*M)* ≥ 15%. The third observation is that, no matter how many different transcript isoforms a gene has, if we focus only on two of them, such as the two with opposing function, one will always be significantly dominant [3–11]. The frequency distribution of *f(1*,*2)* (Fig 4A) shows that for any two isoforms from the same gene, the possibility of the dominant isoform having frequency ≥80% is greater than 73%. This explains how cells can maintain the dominancy of one transcript isoform over others including those with opposing functions. Essentially, our results (Fig 4B) demonstrate that the frequency distribution of the second-most dominant transcript isoform changes with *M*; thus, a more rational way to define the minor transcript isoform may be to use an *M*-related dynamic threshold based on the distribution of *f(2*,*M)*.

## Discussion

In conclusion, we have derived a mathematical model that describes AS based on its physical process. Alternative splicing is a very complex biological process, and many factors contribute to the splicing of a pre-mRNA segment, such as strength of binding of AS complex on splice sites, co-transcriptional splicing, histone modifications on chromatin, poison exons, non-sense mediated degradation (NMD) and so on. Our model only considers the most important of these factors, the strength of splice site binding, and disregards all other factors. In this sense, it is a simple model which nevertheless succeeds very well in explaining many observation regarding AS. AS in our model and in this manuscript refers to the biological process that splices the same pre-mRNA into different transcript isoforms. It covers all five basic modes of alternative splicing: exon skipping or cassette exon, mutually exclusive exons, alternative donor site (alternative 5’ splice site), alternative acceptor site (alternative 3’ splice site) and intron retention. Our model suggests that: (1) AS is a stochastic process such that the relative expression level of different transcript isoforms from the same gene is probabilistically determined by the binding energy of splicing factors at their splice sites; (2) the expression levels of transcript isoforms of a gene follow the Weibull distribution *W(0*.*39*, *b)*, here *b* is a scale parameter dependent on the expression level of the gene, and the scaled expression levels of different transcript isoforms from all genes follow the same Weibull distribution *W(0*.*39)*; and (3) the frequency distributions of all transcript isoforms can be calculated from the Monte Carlo simulation of the Weibull distribution *W(0*.*39)*. This indicates that the expression of a transcript isoform is not a deterministic event but rather a stochastic event, and the detection of a transcript isoform in an RNA-seq dataset depends on both the expression level of its related gene and sequencing depth. We found a simple formula to describe the median frequency of each transcript isoform. Our analysis also provides transcriptome-wide evidence that the dominance rank of transcript isoforms is altered by distinct external signals and identifies 540 genes that switch their major transcript isoform usage in all four T cell subsets studied. Additionally, our analysis reveals that the AS process has an intrinsic tendency to maintain the dominancy of one transcript isoform over others including those with opposing function. Finally, by incorporating previously unexplained observations, the application of our model to describing the statistical distributions of scaled expression level and frequency of transcript isoforms provides a theoretical foundation for understanding the principles that govern relative transcript isoform generation, which in turn regulates cell identity, function and fate.

## Materials and methods

### Ethics statement

Nine healthy study volunteers were recruited through the NIH Department of Transfusion Medicine and gave informed consent for leukapheresis. The study was approved by the NIH Institutional Review Board. Leukaphereses were performed at the NIH Blood Bank, followed by immediate isolation of PBMC by density gradient centrifugation.

### Preparation of peripheral blood CD4 T cells

CD4 T cells were then isolated using the CD4^+^ T Cell Isolation Kit II (Miltenyi), counted, and viably cryopreserved in a freeze medium containing 10% DMSO and 90% sterile filtered, heat inactivated fetal calf serum. Viably cryopreserved peripheral blood CD4 T cells were thawed and stained with ViViD viability dye (Molecular Probes) and fluorescently-labeled monoclonal antibodies against cell surface markers. Staining antibodies included CD3-H7-Allophycocyanin (H7-APC; BD), CD27-Cyanin5-Phycoerythrin (Cy5-PE; Coulter), CD45RO-Texas Red-PE (Coulter), CCR7-Alexa680 (Pharmingen), CD8-Quantum dot-655 (QD655; Invitrogen), CD4-Quantum dot-605 (QD605; Invitrogen), CD19-Pacific Blue (Invitrogen), and CD14-Pacific Blue (Invitrogen). After excluding non-viable cells and those expressing CD19 and CD14, all viable CD3+CD4+CD8- events were gated to collect T_N_ (CD27+CD45RO-), T_CM_ (CD27+CD45RO+CCR7+), T_TM_ (CD27+CD45RO+CCR7-) and T_EM_ (CD27-CD45RO+) populations (S1 Table). Cells were sorted at 4°C and collected in sterile filtered, heat inactivated fetal calf serum. Sorted CD4 T_N_, T_CM_, T_TM_, and T_EM_ subsets were divided into two equal portions to allow comparison between stimulated and unstimulated conditions. Unstimulated portions were immediately subjected to nucleic acid extraction. For stimulation cultures, cells were sedimented at 420g for 7 minutes at 4°C and resuspended in complete culture medium (RPMI 1640 + 10% heat inactivated, sterile filtered calf serum + Penicillin/Streptomycin/Glutamine). They were then combined with T cell activation/expansion beads (anti-CD3/anti-CD2/anti-CD28; Miltenyi) at a 1:2 bead:cell ratio at a final concentration of 2 x 10^6^ cells/mL and incubated at 37°C for 5–6 hours. Following this incubation, stimulated CD4 T cell subsets were subjected to nucleic acid extraction. Cell subsets were lysed in RNAzol RT reagent (Molecular Research Centers) and homogenized by pipetting. Total RNA was then extracted according to the manufacturer’s instructions. Extracted RNA in pellet form was dissolved in RNAse-free water and used for mRNA library construction.

### Sequencing

Sequencing libraries were prepared and sequenced as previously described [30]. In brief, total RNA was enriched for polyadenylated species by two sequential rounds of binding to oligo-dT dynabeads (Life Technologies), chemically fragmented in the presence of Mg^2+^, and reverse transcribed using Superscript III reverse transcriptase (Life Technologies). Second strand cDNA synthesis, end repair, A-tailing, and sequencing adaptor ligation were performed using NEBNext enzyme modules (New England Biolands). Libraries were amplified using universal and indexed primers from the NEBNext system with Kapa 2x Hot Start Readymix (Kapa Biosystems). Amplified libraries were size-selected using Beckman-Coulter Ampure XP beads, quantified by qPCR using the Kapa Library Quantification Kit for Illumina (Kapa Biosystems), and checked for sizing by electrophoresis on a BioAnalyzer (Agilent). Completed libraries were loaded on Illumina Truseq Paired-End v2 Cluster Kits and sequenced in 2 x 100 base paired-end runs on an Illumina HiSeq 2000 sequencer. The final dataset comprised 1.27×10^9^ reads pairs in total, with each cell condition corresponding to 1.59×10^8^ reads pairs and each sample corresponding to 1.76×10^7^ reads pairs on average.

### Sequence analysis

Trimmomatic (version 0.22) was used to remove adapters and low quality bases [31]. The trimmed paired-end reads were mapped to the reference human genome (Hg19) using Tophat (version 2.0.8) and assembled with Cufflinks (version 2.2.1) [32–34]. Cuffmerge was used to merge all novel assemblies and the known human gene set (Ensembl “Homo_sapiens.GRCh37.74.gtf”) to create a merged non-redundant transcript annotation. Finally, Cuffdiff was used to evaluate the expression of genes and their transcript isoforms. All genes with FPKM>1 are included in our analysis. To prove our results are software independent, another software, Salmon (version 0.8.2) was also used to evaluate the expression of genes and their transcript isoforms [26].

### Euclidian distance of two frequency matrix

Supposing *f*1 and *f*2 are two 9×30 frequency matrices, where an element in row *k* and column *M* represents the median frequency of the *k*th most dominant transcript isoform of gene with *M* isoforms, *k*≤*M*. The frequency matrix data may be derived from experimental RNA-seq data, formula (4) or from a simulation of the Weibull distribution *W(0*.*39)*, such as in Fig 2B. The Euclidian distance of the two matrixes can be calculated from following formula,

$$distance\left(f1,f2\right)=\sqrt{\sum_{k=1}^{9}\sum_{M=k}^{30}{\left(f1\left(k,M\right)-f2\left(k,M\right)\right)}^{2}}$$

### Kullback-Leibler divergence of two distributions

Supposing P and Q are two probability distributions, P is from experimental data, Q is from simulated data of Weibull distribution, the Kullback-Leibler divergence (KLd) between P and Q is defined by following formula,

$$KLd\left(P∥Q\right)\;=\;\int_{-\infty }^{\infty }p\left(x\right)log\frac{p\left(x\right)}{q\left(x\right)}dx$$

When P and Q are discrete probability distributions,

$$KLd\left(P∥Q\right)=\sum_{i}P\left(i\right)log\frac{P\left(i\right)}{Q\left(i\right)}$$

KLd represents the amount of information lost when Q is used to approximate P.

### The information content of a distribution

The information content or entropy of a distribution P is defined as,

$$Entropy\left(P\right)\;=\;\int_{-\infty }^{\infty }p\left(x\right)\text{log}\left(p\left(x\right)\right)dx$$

When P is a discrete probability distribution,

$$Entropy\left(P\right)=\underset{i}{\sum }P\left(i\right)\text{log}\left(P\left(i\right)\right)$$

The KLd and entropy in this study are calculated by the KL.plugin function in the R “entropy” package.

### Simulated RNA-seq data

First, we extracted transcript isoform sequences for all human genes from the reference genome (Hg19) according to the Ensembl annotation (Ensembl “Homo_sapiens.GRCh37.74.gtf”). For a gene with M isoforms, we randomly extracted M values from N(20,2) or W(0.39,10) as their expression levels E and proceeded thus: for a transcript isoform with length L and expression level E, we randomly extracted R = int(E*L/100/2+0.5) read pairs to uniformly cover the transcript isoform. Each read pair has 100bp on each end and an average insert length of 100bp. This ensured that the transcript isoform had an expression level of E. We then added reads info and quality info for each read pair. We repeated this process for all transcript isoforms to create simulated FASTQ files. The whole process was repeated ten times to create ten different sequence data for the Normal distribution N(20,2) and the Weibull distribution W(0.39,10), respectively.

### Mathematical estimation and deduction of Weibull distribution

We estimated the value of parameters *a* (0.44) and *b* (0.6) by curve fitting, which is not accurate due to bias in the estimation of the scale factor for each gene as shown below. To illustrate why the shape parameter computed from curve fitting is inaccurate, we performed the same scale transformation on the simulated dataset. The expression values in the simulated dataset strictly follow the Weibull distribution *W(0*.*39*,*1)* as they are produced from this distribution. However, the scaled expression values do not follow the Weibull distribution, and their range is from 0 to M (S10 Fig).

$$scale\left(x_{k}\right)=\frac{x_{k}}{\left(\frac{\sum_{i=1}^{n}x_{i}}{n}\right)}=n\times \frac{x_{k}}{\sum_{i=1}^{n}x_{i}}\leq n$$

The scaled expression is always 1 when *M* = 1. It has two peaks at 0 and 2 when *M* = 2. The larger the *M*, the closer the scaled expression (black histogram) and original expression (red curve). The difference becomes small when *M* = 5. The most distinct difference lies in the maximal value. For original expression, there is no upper bound for the maximal value although the higher the expression the less chance it appears. For scaled expression, the maximal value is bound by *M*. Traditionally, the Weibull plot is used to calculate the shape parameter of the Weibull distribution [24,25]. However, this method cannot be applied here for two reasons. First, the scale parameter and distribution is different for each gene and each condition. Second, the isoform number is limited for each gene, and there is bias in the estimation of the scale parameter for each gene. The comparison of the Weibull plot between the scaled and original values from same simulated data shows that the larger the *M*, the closer the values (S11 Fig). This indicates that we may use genes with a large *M* to calculate the shape parameter. However, the larger the *M*, the fewer the genes with that number of different transcript isoforms.

Since the simulation from the Weibull distribution *W(a = 0*.*39)* explains the frequency distribution of each *f(k*,*M)*, it is reasonable to try to deduce *mf(k*,*M)* and the distribution of *f(k*,*M)* by pure mathematical theoretical deduction. A theoretical deduction requires the distribution of sums of random variables from the Weibull distribution. Unfortunately, there are currently no approximation formulae that describe the distribution of sums of Weibull random variables [35]. This renders it impossible to find a closed form formula to describe the distribution of each *f(k*, *M)*. It is similarly impossible to obtain formula (4) from Weibull model by theoretical deduction.

### Additional information

Supplemental Information includes 11 figures and four tables can be found with this article online.

## Supporting information

S1 Fig Comparison between experimental data and simulated data from Weibull distribution. (A) W(a = 0.2). (B) *W*(a = 0.6). *k* is the rank of transcript isoform. *M* is the number of transcript isoforms of genes. The blue curve represents median values calculated from the approximation formula (4) and the red curve represents median values from simulation of the Weibull distribution.
(TIF)

S2 Fig The frequency distribution of the *k*th dominant transcript isoform. (A) k = 3. (B) *k = 4*. *k* is the rank of transcript isoform. *M* is the number of transcript isoform for a gene. Black curves represent frequency distribution of experimental RNA-seq data. Red curve represents the frequency distribution of simulated data from Weibull distribution *W*(0.39). KLd is the Kullback-Leibler divergence between the two distributions.
(TIF)

S3 Fig The boxplot distribution of transcript isoform frequency *f(k*, *M)* with fixed *k* and increasing *M*. *k* is the rank of transcript isoform. *M* is the number of transcript isoforms of genes. The blue curve represents median values calculated from the approximation formula (4) and the red curve represents median values from simulation of the Weibull distribution *W*(0.39). Boxplot represents frequency distribution calculated from T cell RNA-seq data by **Salmon** (version 0.8.2). The Euclidian distance between the median of box plot and blue curve is 0.182.
(TIFF)

S4 Fig Simulated RNA-seq data with expression levels sampled from the Normal distribution N(20,2). (A) Distribution of expression levels of transcription isoforms. (B) The boxplot distributions of transcript isoform frequency *f(k*, *M)* with fixed *k* and increasing *M*. *k* is the rank of transcript isoform. *M* is the number of transcript isoforms of genes. The blue curves represent median values calculated from the approximation formula (4) and the red curves represent median values from simulation of the Weibull distribution *W*(0.39). Boxplots represent the frequency distribution calculated from simulated RNA-seq data with transcript isoform expression level following a Normal distribution N(20,2). The mode of the expression level is around 5 but not 20 since the expression level have been normalized by the total number of mapped reads pairs and transcript length.
(TIF)

S5 Fig Simulated RNA-seq data with expression levels sampled from the Weibull distribution W(0.39,10). (A) Distribution of expression levels of transcription isoforms. (B) The boxplot distributions of transcript isoform frequency *f(k*, *M)* with fixed *k* and increasing *M*. *k* is the rank of transcript isoform. *M* is the number of transcript isoforms of genes. The blue curves represent median values calculated from the approximation formula (4) and the red curves represent median values from simulation of the Weibull distribution *W*(0.39). Boxplots represent the frequency distribution calculated from simulated RNA-seq data with transcript isoform expression level following a Weibull distribution W(0.39,10).
(TIF)

S6 Fig The boxplot distribution of transcript isoform frequency *f(k*, *M)* with fixed *k* and increasing *M*. (A) Leukemia K562. (B) Breast cancer MCF-7 cell line. *k* is the rank of transcript isoform. *M* is the number of transcript isoforms of genes. The blue curve represents median values calculated from the approximation formula (4) and the red curve represents median values from simulation of the Weibull distribution *W*(0.39). Boxplot represents frequency distribution calculated from RNA-seq data.
(TIF)

S7 Fig The boxplot distribution of transcript isoform frequency *f(k*, *M)* with fixed *k* and increasing *M*. (A) Colorectal cancer (GSE50760). (B) Embryonic stem cell (GSE60178). *k* is the rank of transcript isoform. *M* is the number of transcript isoforms of genes. The blue curve represents median values calculated from the approximation formula (4) and the red curve represents median values from simulation of the Weibull distribution *W*(0.39). Boxplot represents frequency distribution calculated from RNA-seq data.
(TIF)

S8 Fig The boxplot distribution of transcript isoform frequency *f(k*, *M)* with fixed *k* and increasing *M*. *k* is the rank of transcript isoform. *M* is the number of transcript isoforms of genes. The blue curve represents median values calculated from the approximation formula (4) and the red curve represents median values from simulation of the Weibull distribution *W*(0.39). The result is based on the Ensembl gene set (Ensembl “Homo_sapiens.GRCh37.74.gtf”) and our own RNA-seq data.
(TIFF)

S9 Fig The gene ontology biological processes enriched in the 540 gene switching their most dominant transcript isoform between resting and activated status for all four T cell subsets. (TIF)

S10 Fig The distribution of the scaled expression level of transcript isoforms of simulated RNA-seq data from Weibull distribution *W*(0.39). Histograms represent the distribution of the scaled value. Red curves represent the original value before scaling, which is the probability density function of *W*(0.39).
(TIF)

S11 Fig Weibull plot of simulated RNA-seq data from Weibull distribution *W*(0.39). Red curves represent the original value and black curves represent the scaled value.
(TIF)

S1 Table Summary of sequence files. We collected the samples from nine patients. For each patient, we sequenced four types of T cells: Naïve (T_N_, CD27+CD45RO-), Central Memory (T_CM_, CD27+CD45RO+CCR7+), Transitional Memory (T_TM_, CD27+CD45RO+CCR7-), and Effector Memory (T_EM_, CD27-CD45RO+). Each type of T cell has two states, “Rest” (Resting, unstimulated) and “Activ” (Stimulated by a global T cell activation reagent). We sequenced 72 samples in total.
(DOCX)

S2 Table Frequency threshold of significantly dominant transcript isoform for genes with different isoform number. (DOCX)

S3 Table Additional 18 RNA-seq datasets that were analyzed. (DOCX)

S4 Table Change of the most dominant transcript isoform between resting and activated condition based on the Ensembl gene set. 540 AS genes switch their most dominant isoform between the resting and activated conditions for all four subsets of T cells (dark gray shaded). Another 891 AS genes switch the most dominant isoform between resting and activated conditions for three of four subsets of T cells.
(DOCX)
