# Supplementary figures and images for: Stochastic principles governing alternative splicing of RNA
Source: PLoS Comput Biol. 2017 Sep 14;13(9):e1005761. doi: 10.1371/journal.pcbi.1005761 (PMC5614656; doi:10.1371/journal.pcbi.1005761)

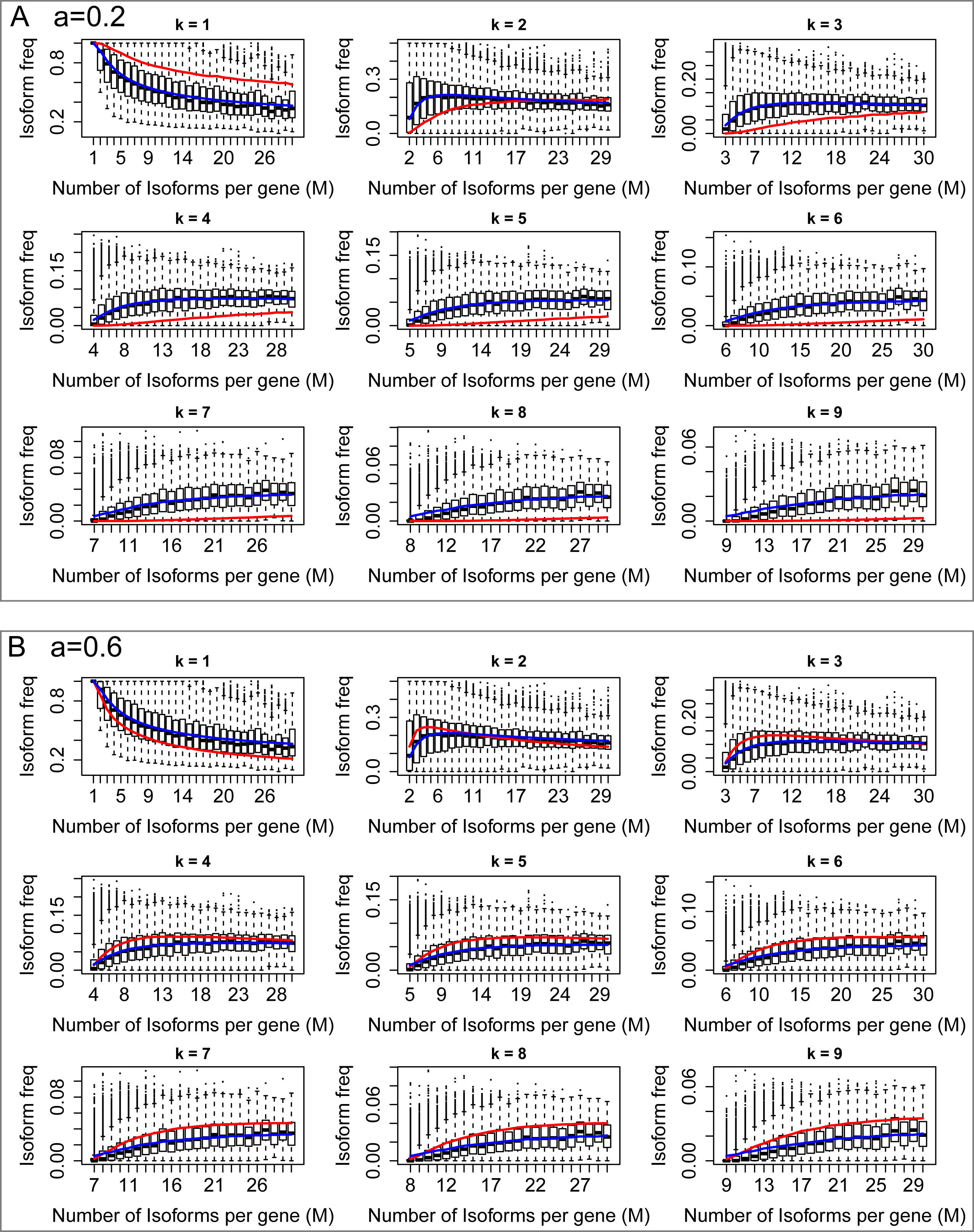

Supplement: S1 Fig — (A) W(a = 0.2). (B) W(a = 0.6). k is the rank of transcript isoform. M is the number of transcript isoforms of genes. The blue curve represents median values calculated from the approximation formula (4) and the red curve represents median values from simulation of the Weibull distribution. (TIF) [file pcbi.1005761.s001.tif]

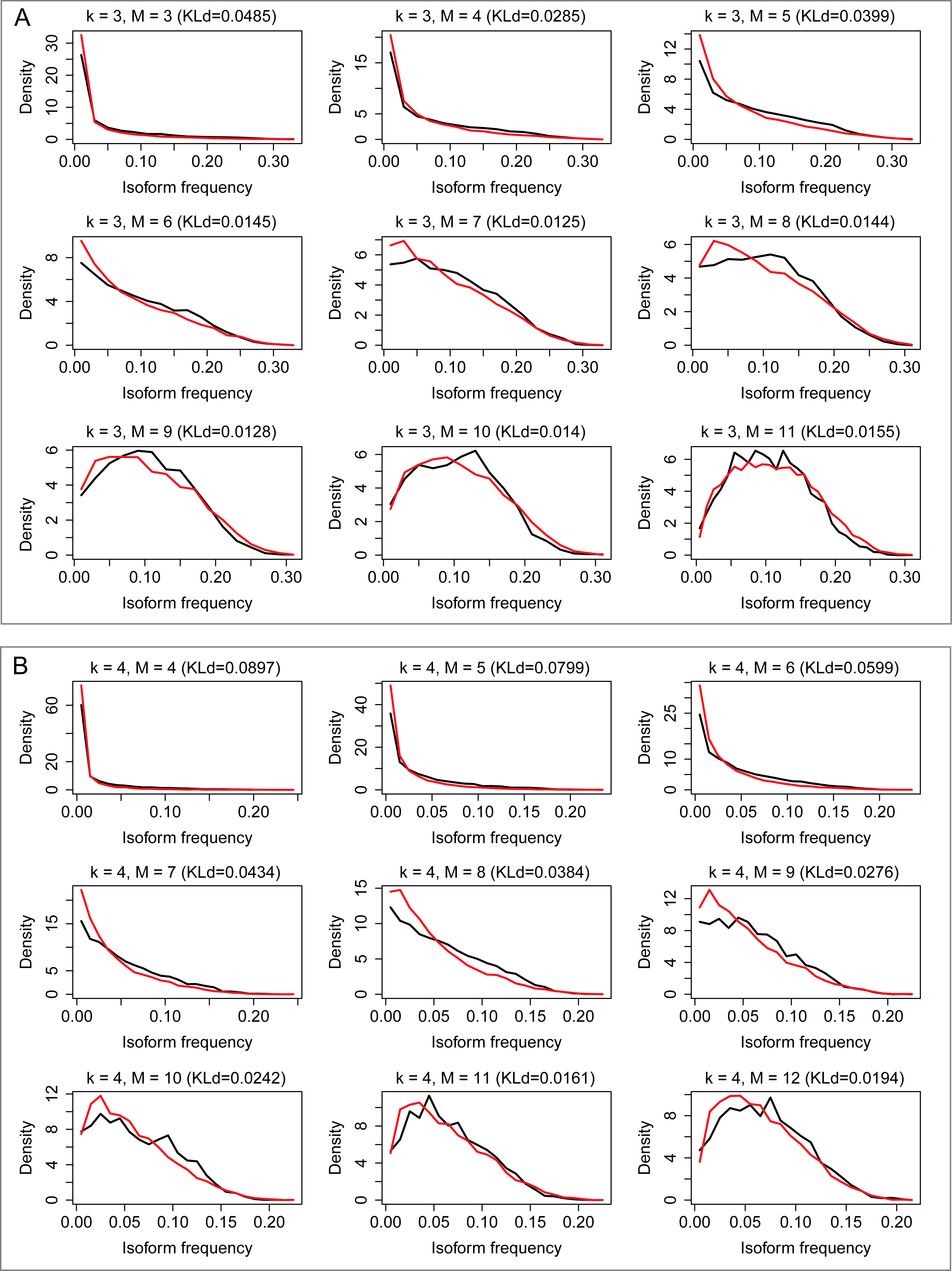

Supplement: S2 Fig — (A) k = 3. (B) k = 4. k is the rank of transcript isoform. M is the number of transcript isoform for a gene. Black curves represent frequency distribution of experimental RNA-seq data. Red curve represents the frequency distribution of simulated data from Weibull distribution W(0.39). KLd is the Kullback-Leibler divergence between the two distributions. (TIF) [file pcbi.1005761.s002.tif]

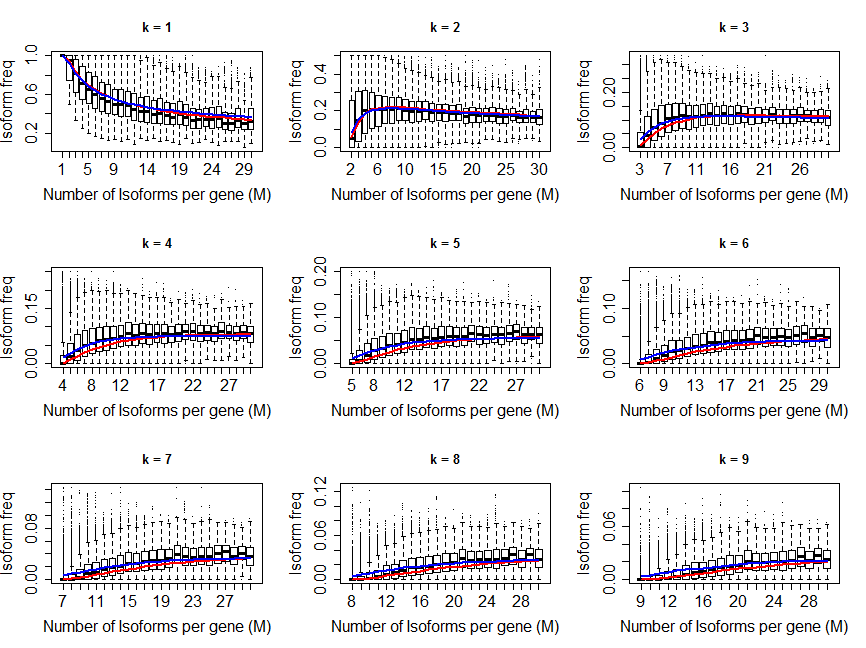

Supplement: S3 Fig — k is the rank of transcript isoform. M is the number of transcript isoforms of genes. The blue curve represents median values calculated from the approximation formula (4) and the red curve represents median values from simulation of the Weibull distribution W(0.39). Boxplot represents frequency distribution calculated from T cell RNA-seq data by Salmon (version 0.8.2). The Euclidian distance between the median of box plot and blue curve is 0.182. (TIFF) [file pcbi.1005761.s003.tiff]

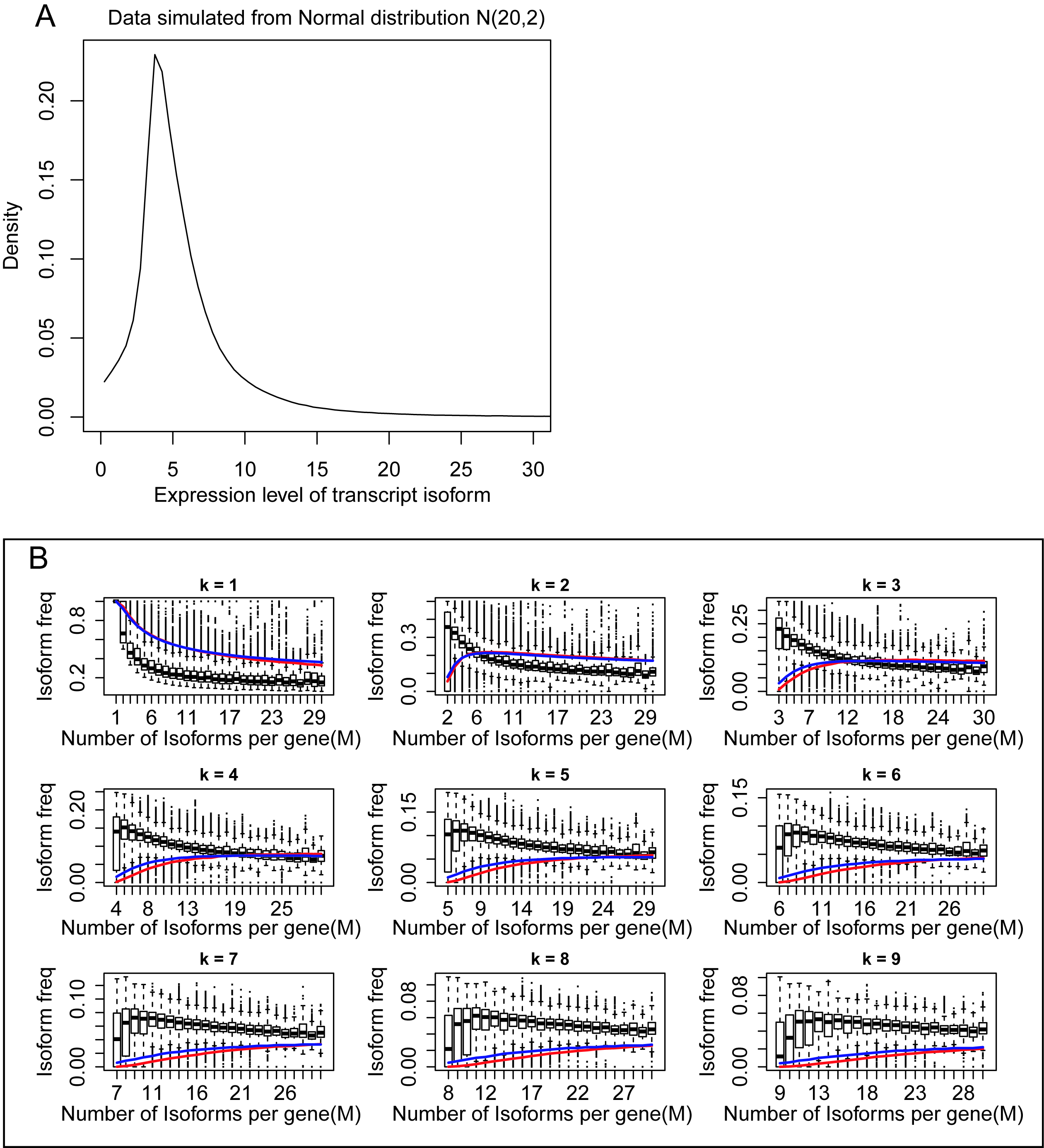

Supplement: S4 Fig — (A) Distribution of expression levels of transcription isoforms. (B) The boxplot distributions of transcript isoform frequency f(k, M) with fixed k and increasing M. k is the rank of transcript isoform. M is the number of transcript isoforms of genes. The blue curves represent median values calculated from the approximation formula (4) and the red curves represent median values from simulation of the Weibull distribution W(0.39). Boxplots represent the frequency distribution calculated from simulated RNA-seq data with transcript isoform expression level following a Normal distribution N(20,2). The mode of the expression level is around 5 but not 20 since the expression level have been normalized by the total number of mapped reads pairs and transcript length. (TIF) [file pcbi.1005761.s004.tif]

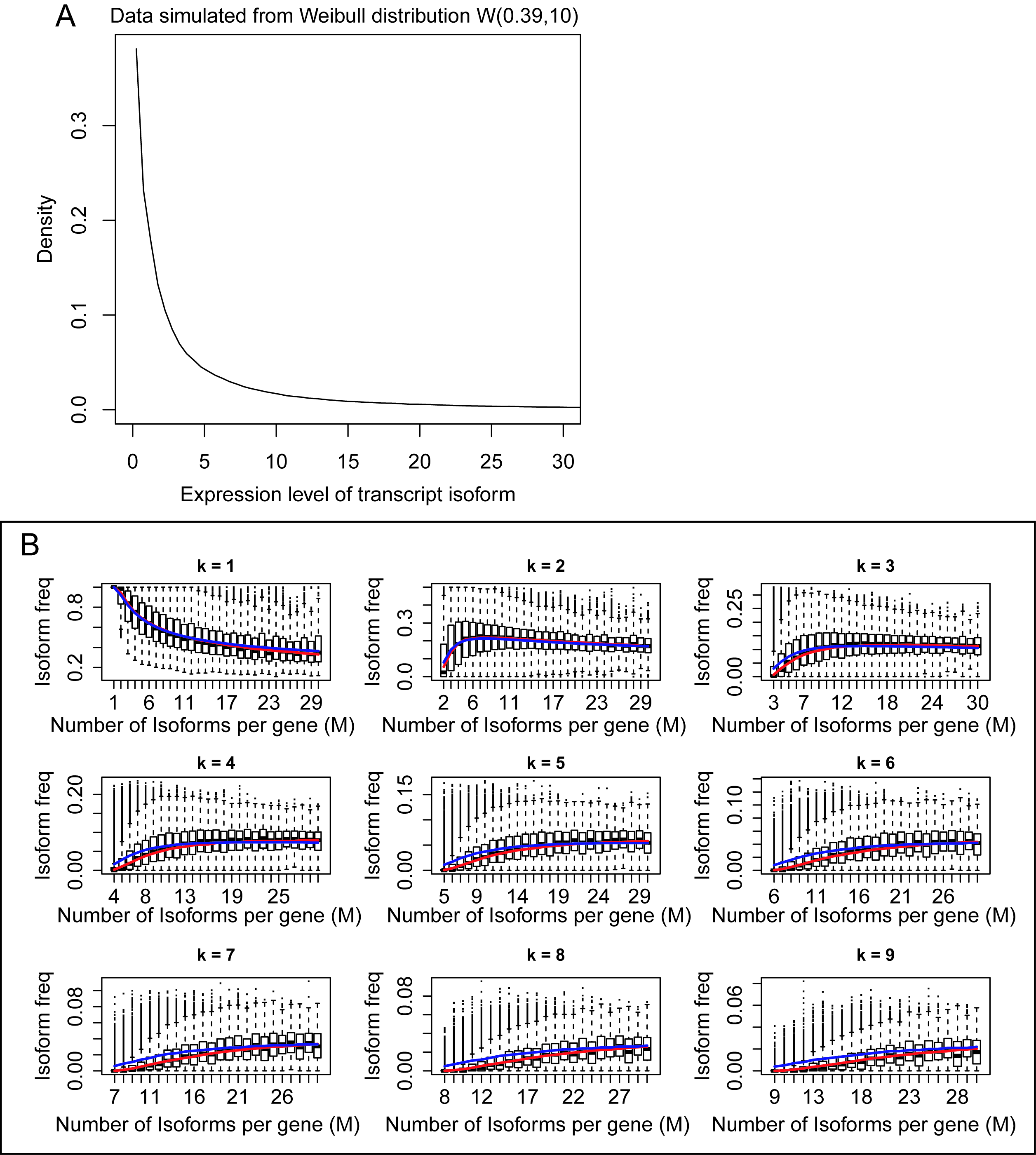

Supplement: S5 Fig — (A) Distribution of expression levels of transcription isoforms. (B) The boxplot distributions of transcript isoform frequency f(k, M) with fixed k and increasing M. k is the rank of transcript isoform. M is the number of transcript isoforms of genes. The blue curves represent median values calculated from the approximation formula (4) and the red curves represent median values from simulation of the Weibull distribution W(0.39). Boxplots represent the frequency distribution calculated from simulated RNA-seq data with transcript isoform expression level following a Weibull distribution W(0.39,10). (TIF) [file pcbi.1005761.s005.tif]

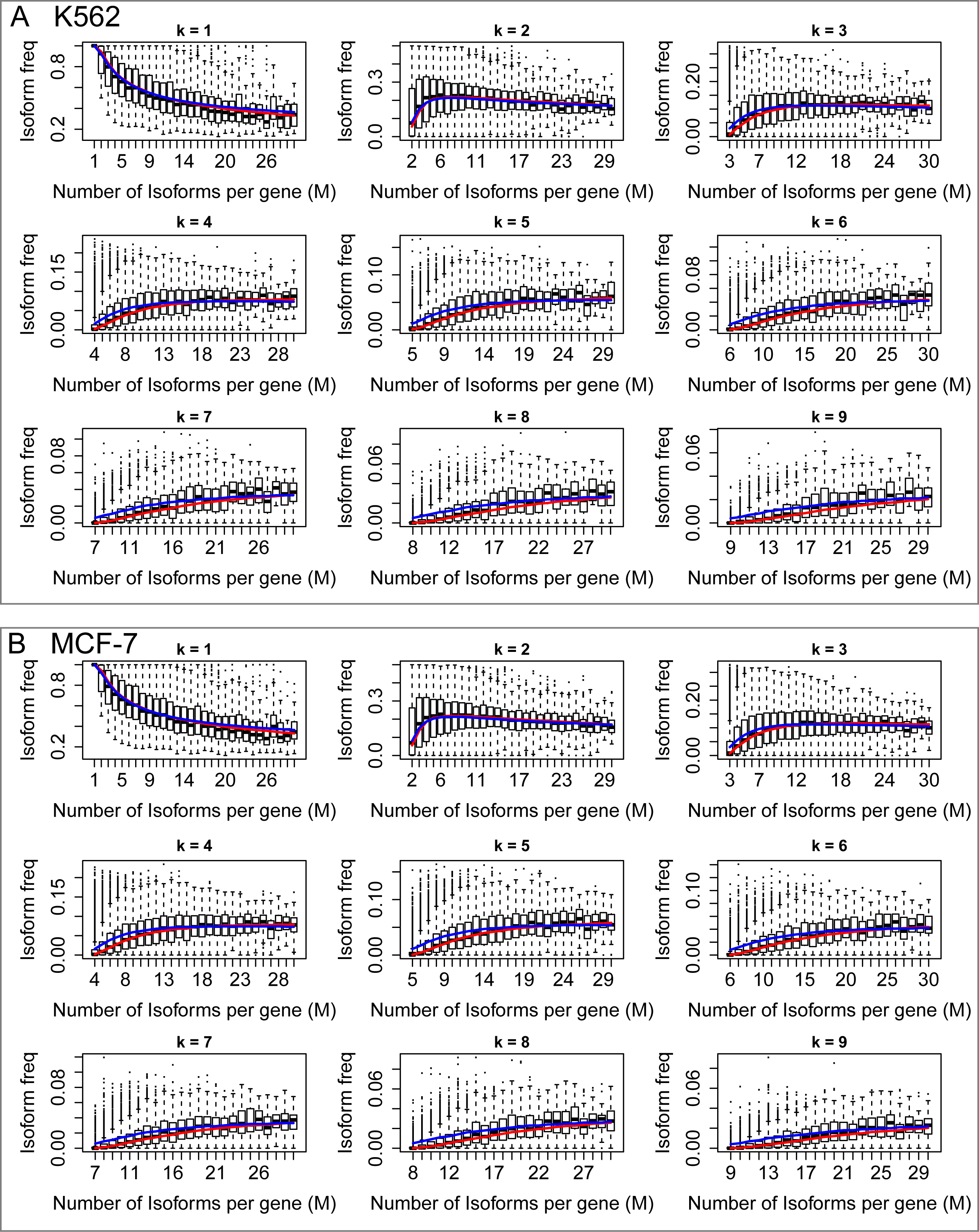

Supplement: S6 Fig — (A) Leukemia K562. (B) Breast cancer MCF-7 cell line. k is the rank of transcript isoform. M is the number of transcript isoforms of genes. The blue curve represents median values calculated from the approximation formula (4) and the red curve represents median values from simulation of the Weibull distribution W(0.39). Boxplot represents frequency distribution calculated from RNA-seq data. (TIF) [file pcbi.1005761.s006.tif]

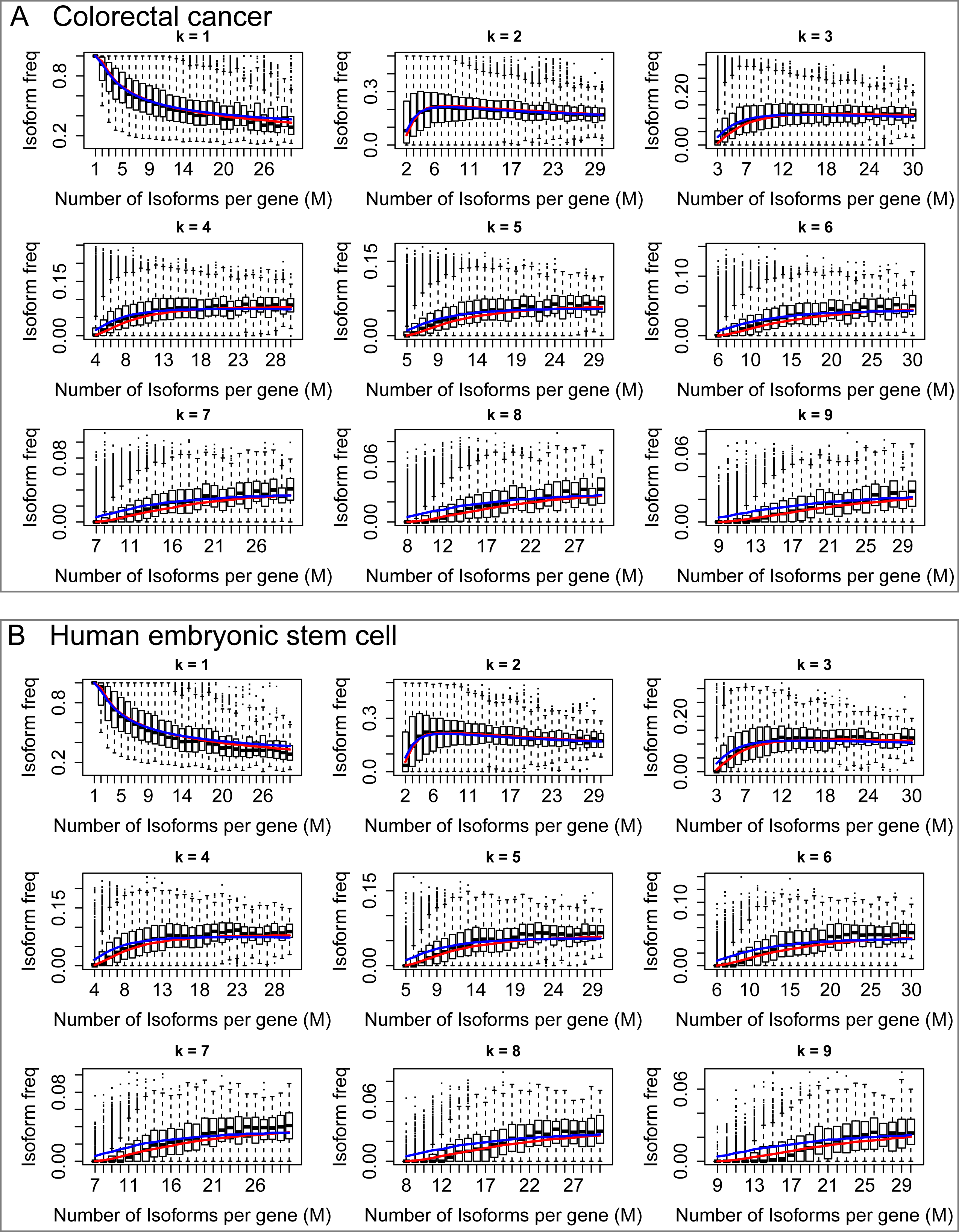

Supplement: S7 Fig — (A) Colorectal cancer (GSE50760). (B) Embryonic stem cell (GSE60178). k is the rank of transcript isoform. M is the number of transcript isoforms of genes. The blue curve represents median values calculated from the approximation formula (4) and the red curve represents median values from simulation of the Weibull distribution W(0.39). Boxplot represents frequency distribution calculated from RNA-seq data. (TIF) [file pcbi.1005761.s007.tif]

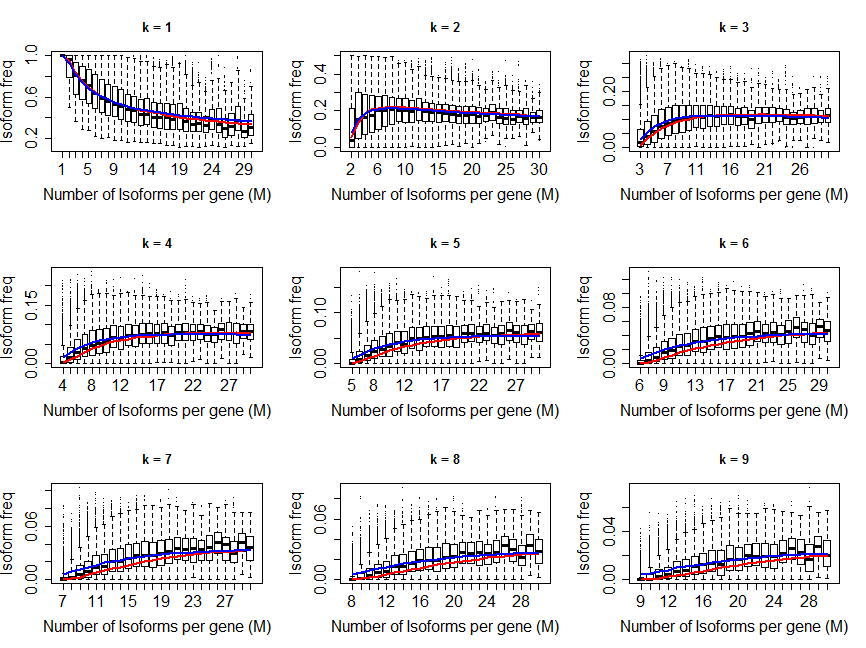

Supplement: S8 Fig — k is the rank of transcript isoform. M is the number of transcript isoforms of genes. The blue curve represents median values calculated from the approximation formula (4) and the red curve represents median values from simulation of the Weibull distribution W(0.39). The result is based on the Ensembl gene set (Ensembl “Homo_sapiens.GRCh37.74.gtf”) and our own RNA-seq data. (TIFF) [file pcbi.1005761.s008.tiff]

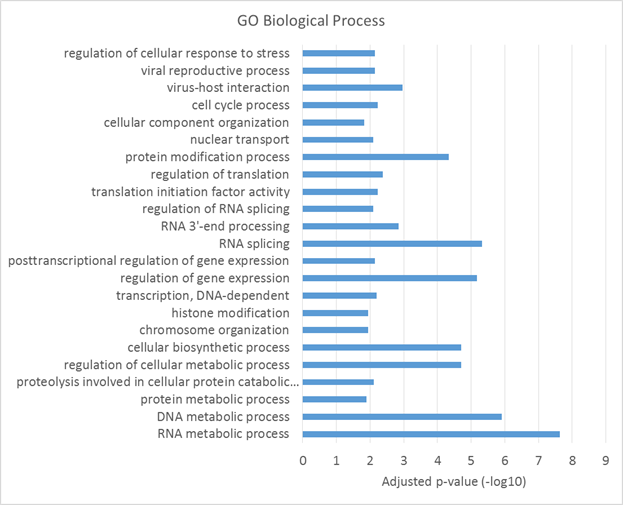

Supplement: S9 Fig — (TIF) [file pcbi.1005761.s009.tif]

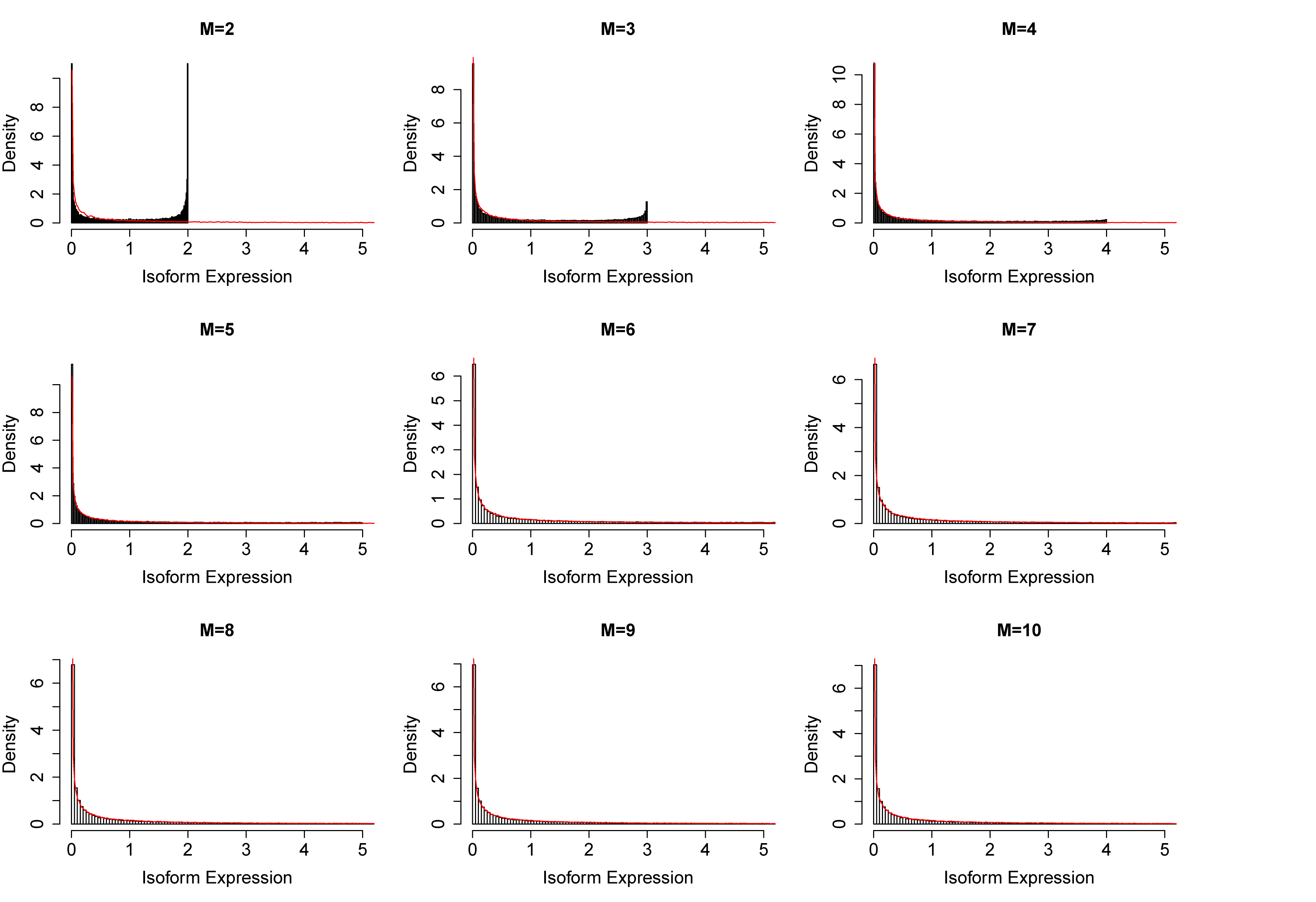

Supplement: S10 Fig — Histograms represent the distribution of the scaled value. Red curves represent the original value before scaling, which is the probability density function of W(0.39). (TIF) [file pcbi.1005761.s010.tif]

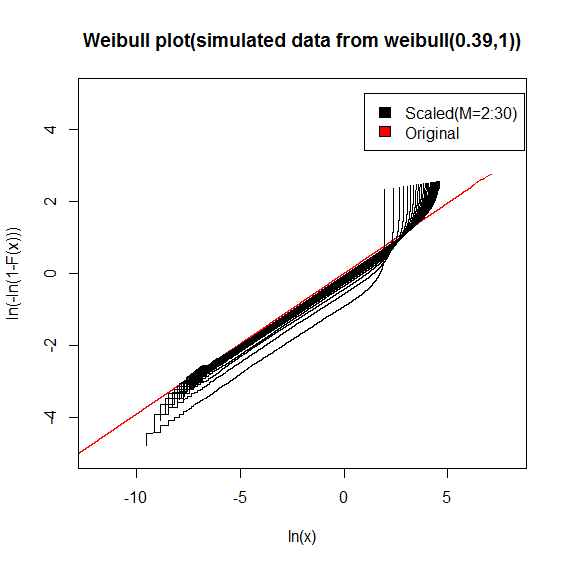

Supplement: S11 Fig — Red curves represent the original value and black curves represent the scaled value. (TIF) [file pcbi.1005761.s011.tif]
