# Supplementary material for: Stochastic principles governing alternative splicing of RNA
Source: PLoS Comput Biol. 2017 Sep 14;13(9):e1005761. doi: 10.1371/journal.pcbi.1005761 (PMC5614656; doi:10.1371/journal.pcbi.1005761)
Supplement: S1 Table — We collected the samples from nine patients. For each patient, we sequenced four types of T cells: Naïve (TN, CD27+CD45RO-), Central Memory (TCM, CD27+CD45RO+CCR7+), Transitional Memory (TTM, CD27+CD45RO+CCR7-), and Effector Memory (TEM, CD27-CD45RO+). Each type of T cell has two states, “Rest” (Resting, unstimulated) and “Activ” (Stimulated by a global T cell activation reagent). We sequenced 72 samples in total. (DOCX) [file pcbi.1005761.s012.docx]

**S1 Table**. Summary of sequence files. We collected the samples from nine patients. For each patient, we sequenced four types of T cells: Naïve (T_N_, CD27+CD45RO-), Central Memory (T_CM_, CD27+CD45RO+CCR7+), Transitional Memory (T_TM_, CD27+CD45RO+CCR7-), and Effector Memory (T_EM_, CD27-CD45RO+). Each type of T cell has two states, “Rest” (Resting, unstimulated) and “Activ” (Stimulated by a global T cell activation reagent). We sequenced 72 samples in total.

| Patient | Naïve | | CM | | TM | | EM | |
| --- | --- | --- | --- | --- | --- | --- | --- | --- |
|  | Activ | Rest | Activ | Rest | Activ | Rest | Activ | Rest |
| 1 | A6_7* | B8_2 | B2_12 | A8_4 | B2_2 | A1_5 | B7_4 | A3_6 |
| 2 | A7_2 | B3_5 | B8_4 | A5_6 | B6_5 | A5_7 | A7_6 | A1_12 |
| 3 | B7_6 | A8_7 | B6_7 | B6_12 | A8_12 | A3_2 | B1_2 | A2_4 |
| 4 | A2_2 | B2_5 | A7_4 | B8_6 | A2_5 | B1_7 | A6_6 | A2_12 |
| 5 | A8_5 | A2_7 | A8_6 | B8_12 | B3_7 | A8_2 | A6_12 | B2_4 |
| 6 | B1_5 | B7_7 | B3_6 | A3_12 | B2_7 | B7_2 | B3_12 | A3_4 |
| 7 | A1_7 | A6_2 | A7_12 | B6_4 | A5_2 | B8_5 | A6_4 | B1_6 |
| 8 | B3_2 | A7_5 | A1_4 | A1_6 | A3_5 | B8_7 | A2_6 | B1_12 |
| 9 | B7_5 | A3_7 | B6_6 | A5_12 | A7_7 | B6_2 | B7_12 | A5_4 |

Note: *, sequence file name. Taking A6_7 as an example, A represents flowcell, 6 represents lane number and 7 represents barcode index.
