# Supplementary material for: Stochastic principles governing alternative splicing of RNA
Source: PLoS Comput Biol. 2017 Sep 14;13(9):e1005761. doi: 10.1371/journal.pcbi.1005761 (PMC5614656; doi:10.1371/journal.pcbi.1005761)
Supplement: S2 Table — (DOCX) [file pcbi.1005761.s013.docx]

**S2 Table**. Frequency threshold of significantly dominant transcript isoform for genes with different isoform number.

| *M* | *p*=0.05 | *p*=0.01 | *M* | *p*=0.05 | *p*=0.01 | *M* | *p*=0.05 | *p*=0.01 |
| --- | --- | --- | --- | --- | --- | --- | --- | --- |
|  |  |  | 11 | 0.483 | 0.781 | 21 | 0.255 | 0.537 |
| 2 | 0.999 | 0.9999 | 12 | 0.449 | 0.75 | 22 | 0.244 | 0.518 |
| 3 | 0.98 | 0.998 | 13 | 0.416 | 0.724 | 23 | 0.232 | 0.507 |
| 4 | 0.923 | 0.988 | 14 | 0.387 | 0.7 | 24 | 0.223 | 0.486 |
| 5 | 0.852 | 0.972 | 15 | 0.361 | 0.669 | 25 | 0.213 | 0.477 |
| 6 | 0.779 | 0.946 | 16 | 0.338 | 0.655 | 26 | 0.204 | 0.462 |
| 7 | 0.7 | 0.917 | 17 | 0.315 | 0.623 | 27 | 0.197 | 0.449 |
| 8 | 0.637 | 0.879 | 18 | 0.299 | 0.592 | 28 | 0.188 | 0.432 |
| 9 | 0.578 | 0.849 | 19 | 0.282 | 0.579 | 29 | 0.18 | 0.424 |
| 10 | 0.529 | 0.814 | 20 | 0.269 | 0.554 | 30 | 0.174 | 0.414 |
