# Supplementary material for: Stochastic principles governing alternative splicing of RNA
Source: PLoS Comput Biol. 2017 Sep 14;13(9):e1005761. doi: 10.1371/journal.pcbi.1005761 (PMC5614656; doi:10.1371/journal.pcbi.1005761)
Supplement: S3 Table — (DOCX) [file pcbi.1005761.s014.docx]

**S3 Table**. Additional 18 RNA-seq datasets that were analyzed.

| GEO Accession | Sample annotation |
| --- | --- |
| GSE33480 | GM12878 cell line (blood, mesoderm,normal) |
|  | GM12891 cell line (blood, mesoderm) |
|  | GM12892 cell line (GM12892) |
|  | H1-hESC cell line (embryonic stem cells) |
|  | HCT-116 cell line (colorectal carcinoma) |
|  | HeLa-S3 cell line (cervical carcinoma) |
|  | HepG2 cell line (liver carcinoma) |
|  | HSMM cell line (normal human Skeletal muscle myoblasts) |
|  | HUVEC cell line (umbilical vein endothelial cells) |
|  | K562 cell line (leukemia) |
|  | LHCN-M2 cell line (skeletal myoblasts) |
|  | MCF-7 cell line (breast cancer) |
|  | NHEK cell line (epidermal keratinocytes) |
|  | NHLF cell line (normal human lung fibroblasts) |
| GSE33294 | Human hepatocellular carcinoma |
| GSE50760 | Colorectal cancer |
| GSE52194 | Breast cancer |
| GSE60178 | Human embryonic stem cell |
