# Supplementary material for: Stochastic principles governing alternative splicing of RNA
Source: PLoS Comput Biol. 2017 Sep 14;13(9):e1005761. doi: 10.1371/journal.pcbi.1005761 (PMC5614656; doi:10.1371/journal.pcbi.1005761)
Supplement: S4 Table — 540 AS genes switch their most dominant isoform between the resting and activated conditions for all four subsets of T cells (dark gray shaded). Another 891 AS genes switch the most dominant isoform between resting and activated conditions for three of four subsets of T cells. (DOCX) [file pcbi.1005761.s015.docx]

**S4 Table**. Change of the most dominant transcript isoform between resting and activated condition based on the Ensembl gene set. 540 AS genes switch their most dominant isoform between the resting and activated conditions for all four subsets of T cells (dark gray shaded). Another 891 AS genes switch the most dominant isoform between resting and activated conditions for three of four subsets of T cells.

| Pattern | # Gene | Pattern | # Gene | Pattern | # Gene |
| --- | --- | --- | --- | --- | --- |
| ABABABAB | 540 | ABBABABA | 23 | ABABABBC | 5 |
| AABABABA | 155 | ABCBCBCB | 19 | ABBCABAB | 4 |
| ABABABAA | 140 | ABACACAC | 18 | ABCAABAB | 4 |
| AAABABAB | 83 | ABABCBAB | 17 | ABABABCC | 4 |
| ABAAABAB | 61 | ABCBABAB | 14 | ABABCAAB | 4 |
| ABABAAAB | 55 | ABABABBA | 13 | ABABCCAB | 2 |
| ABABABAC | 44 | ABABACAB | 12 | AABCBCBC | 2 |
| ABACABAB | 40 | ABBAABAB | 10 | ABABCDAB | 2 |
| ABABBBAB | 39 | ABCACACA | 9 | ABABBCAB | 2 |
| ABABABBB | 37 | ABABABCD | 6 | ABBCBCBC | 2 |
| ABABABCB | 29 | ABABBAAB | 5 | ABCDABAB | 1 |
| ABBBABAB | 24 | ABABABCA | 5 | ABCDCDCD | 1 |

Note: The eight letters in pattern represent the most dominant isoforms in eight different conditions, “active naive T cell”, “rest naive T cell”, “active central memory T cell”, “rest central memory T cell”, “active transitional memory T cell”, “rest transitional memory T cell”, “active effector memory T cell”, “rest effector memory T cell”. Different letter represents different dominant isoform.
